# Thermal Spin Coated PbS QD SWIR Imager for Non‐Invasive Glucose Monitoring

**DOI:** 10.1002/advs.75944

**Published:** 2026-06-01

**Authors:** Lei Rao, Shuo Cheng, Qian Chen, Jiankai Wang, Jingrui Ma, Junjie Hao, Xiao Wei Sun, Wei Chen, Cun Zheng Ning, Haodong Tang

**Affiliations:** ^1^ College of Integrated Circuits and Optoelectronic Chips Shenzhen Technology University Shenzhen China; ^2^ College of Engineering Physics Shenzhen Technology University Shenzhen China; ^3^ Institute of Nanoscience and Applications, and Department of Electronic and Electrical Engineering Southern University of Science and Technology Shenzhen China

**Keywords:** bandgap, dark current, lead sulfide, materials science, optoelectronics, photodetector, quantum dot, quantum efficiency, responsivity

## Abstract

Colloidal lead sulfide quantum dots are attractive for short‐wave infrared photodetectors due to their tunable bandgap and solution‐process compatibility, yet device performance is often limited by high dark current, inefficient carrier extraction, and poor stability. Here, we report a new strategy of combining thermal spin‐coating and annealing that improves quantum dot film quality by regulating solvent evaporation kinetics and stacking behavior during deposition. Elevating the substrate temperature during spin‐coating induces dense and uniform quantum dot assemblies with reduced trap density and improved interfacial contact. Photodetectors fabricated at an optimized temperature of 65°C exhibit substantially enhanced performance, including a responsivity of 0.765 A/W, a specific detectivity of 3.57 × 10^1^ ^1^ Jones, and a −3 dB bandwidth of 108 kHz, accompanied by over 50% reduction in dark current density. Importantly, the optimized devices show improved long‐term stability, retaining lower dark current and higher external quantum efficiency after prolonged storage without encapsulation. Leveraging these advantages, the photodetectors are further integrated into an imaging array and applied to non‐invasive glucose monitoring using dual‐wavelength ratiometric detection. This work establishes thermal spin‐coating as a simple and scalable route toward high‐performance, stable quantum dot infrared photodetectors for imaging and biomedical sensing applications.

## Graphical abstract

Thermal spin‐coating enables controlled film formation of PbS quantum dots, leading to denser packing, reduced defect‐assisted transport, and improved carrier extraction. The resulting photodetectors exhibit suppressed dark current, enhanced responsivity, and faster response. This simple, scalable strategy provides an effective route toward high‐performance SWIR imaging and integrated sensing applications.

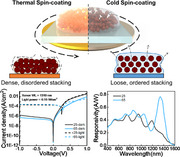

## 1 Introduction

Colloidal lead sulfide (PbS) quantum dots (QDs) have attracted wide attention as next‐generation materials for infrared photodetectors due to their size‐tunable bandgap, high absorption coefficients in the visible to short‐wave infrared (SWIR) regions, and compatibility with solution processing [1, 2, 3, 4, 5, 6]. These properties make PbS QDs highly attractive for emerging applications such as spectrometers, night vision, environmental sensing, and non‐destructive inspection systems [7, 8, 9, 10]. Compared to traditional epitaxial infrared detectors, PbS QD photodetectors offer significant advantages, including low fabrication cost, large‐area scalability, and potential monolithic integration with CMOS readout circuits, opening up new opportunities for industrial deployment in cost‐sensitive fields [11, 12, 13]. Despite their advantages, the practical performance of PbS QD photodetectors remains limited by several intrinsic and processing‐related factors. Among the most significant bottlenecks are the high dark current densities and poor carrier extraction efficiency that are caused by sub‐optimal film morphology and interfacial quality [14]. During solution‐phase deposition, QD layers often suffer from microcracks and voids due to incomplete solvent evaporation or non‐uniform ligand exchange [15]. These structural imperfections not only increase surface roughness but also create localized electric field distortions, leading to trap‐assisted recombination and high leakage current. Another critical issue is the insufficient extraction of photogenerated carriers at the interfaces between the active layer and the transport layers [16]. Due to energy level mismatches or poorly controlled interfacial dipoles, photogenerated carriers can accumulate at energy barriers, resulting in space‐charge buildup and carrier recombination [17]. These effects collectively suppress responsivity, slow down response time, and increase noise current, ultimately reducing the specific detectivity (D*). These limitations severely hinder the application of PbS QD photodetectors in low‐light and high‐speed imaging scenarios [18, 19, 20, 21].

To address these limitations, a variety of strategies have been developed, primarily focused on defect passivation and interfacial engineering [22, 23]. One common approach involves the incorporation of buffer or passivation layers between the QD film and carrier transport layers. For instance, materials such as ZnO, MoO_x_, TiO_2_, or organic semiconductors have been introduced to modify band alignment, enhance carrier selectivity, and suppress interface recombination [24, 25, 26, 27, 28]. Similarly, ligand engineering using inorganic halide ligands or mixed‐ligand systems has been used to improve QD surface passivation and film packing [14, 29]. In parallel, optimization of the carrier transport layers, particularly the PbS‐EDT hole transport layer, has also been explored. Strategies such as mixing QDs of different sizes, implementing freeze‐centrifuged QDs, and fine‐tuning ligand exchange conditions have been shown to significantly improve the film quality and suppress leakage current by minimizing structural defects and enhancing packing uniformity [16, 17, 18, 30]. However, these methods still rely on additional materials or multi‐step processing, which limit their scalability, stability, and compatibility with further CMOS integration.

In contrast to these additive or chemically intensive strategies, process‐driven methods that improve film quality and interface properties through controlled thermal treatments have gained increasing attention. For example, post‐deposition annealing can promote ligand reorganization, improve inter‐dot coupling, and relax residual stress within the film [31]. However, conventional annealing processes are typically decoupled from the film formation stage, and their effectiveness can be highly sensitive to initial film morphology [32, 33, 34]. In this context, thermal spin‐coating annealing, where substrate temperature is elevated during the spin‐coating process, provides an integrated and controllable method to tune film formation kinetics in situ. This process affects solvent evaporation rate, QD assembly dynamics, and inter‐dot packing behavior, which in turn influence film continuity, surface roughness, and defect formation.

Herein, we report a systematic study on the effect of thermal spin‐coating temperature on the performance of PbS QD photodetectors. By tuning the substrate temperature during spin‐coating from room temperature up to 95°C, we identify an optimized processing window (65°C) that yields significant improvements in film morphology and device performance. Grazing‐incidence x‐ray scattering results suggest that simultaneous temperature during deposition increases, QDs stack toward a disordered configuration with a smaller inter‐dot distance and a broader distribution width. Devices fabricated at 65°C exhibit reduced dark current density, suppressed low‐frequency noise, and enhanced detectivity, with responsivity reaching up to 0.765 A/W and D* exceeding 3.5 × 10^1^ ^1^ Jones. Time‐resolved photoluminescence (TRPL) measurements reveal longer carrier lifetimes in films processed at elevated temperatures, indicating reduced trap‐state density. Impedance spectroscopy further confirms improved charge separation and lower recombination rates at the interfaces. Notably, all performance improvements are achieved without the use of additional passivation layers or compositional modification, highlighting the simplicity and scalability of the proposed approach. The process is fully compatible with the standard QD ink fabrication process and does not alter the intrinsic optical properties of the QDs, as confirmed by absorption and photoluminescence spectra across processing conditions. Moreover, compared with room‐temperature spin‐coating, the optimized thermal process maintains film thickness uniformity while significantly enhancing structural and electronic quality.

## 2 Results and Discussion

### 2.1 Stacking of QDs During Thermal and Cold Spin‐Coating

The fabrication of the QD active layer plays a central role in determining the morphology, uniformity, and defect density of the final film, thereby directly impacting device performance. In conventional fabrication processes, a two‐step method is typically employed, consisting of room‐temperature spin‐coating followed by post‐deposition thermal annealing. This method, referred to as cold spin‐coating, allows the QDs to undergo structural reorganization during and after film formation. Specifically, during spin‐coating, centrifugal force distributes the QD solution across the substrate, and only a thin layer remains due to surface tension. As the solvent evaporates, QDs gradually assemble into a solid film. The film formation dynamics are primarily governed by the evaporation rate of the solvent and the concentration of QDs in solution. In the cold spin‐coating process, gradient solvent evaporation at room temperature allows QDs to retain sufficient lateral mobility during film formation. This enables QDs to travel longer distances and form more ordered packing structures across the film. The subsequent thermal annealing step drives further reorganization of QD domains, contributing to improved inter‐dot electronic coupling and partial defect repair. However, the final film structure remains highly dependent on the initial self‐assembly dynamics during the spin‐coating process, which are sensitive to environmental and material parameters.

**FIGURE 1:**
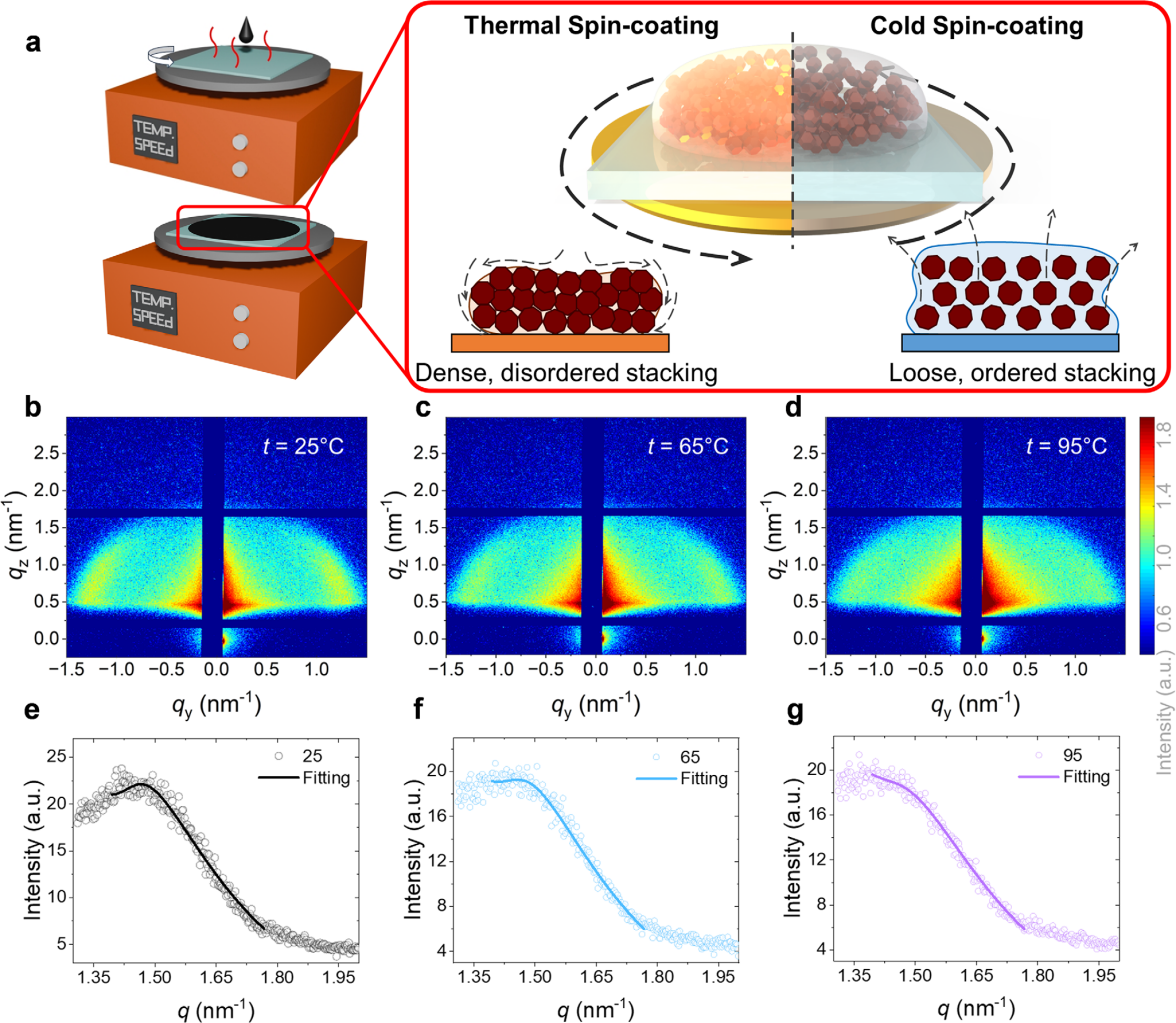
(a) Schematic illustration of thermal spin‐coating and cold spin‐coating processes for PbS QD film formation. In thermal spin‐coating, elevated substrate temperature accelerates solvent evaporation and yields dense, disordered stacking, whereas cold spin‐coating at room temperature leads to loose, more ordered stacking. (b–d) GISAXS 2D patterns of PbS QD films prepared at 25°C, 65°C, and 95°C, respectively, showing the evolution of scattering intensity and inter‐dot correlation with increasing spin‐coating temperature. (e–g) Corresponding in‐plane line cuts and fitting curves extracted from the GISAXS patterns in (b–d), used to determine the effective QD radius and inter‐dot distance, confirming a transition from ordered, loosely packed lattices at 25°C to more compact, less ordered assemblies at higher temperatures.

In contrast, the thermal spin‐coating strategy used in this study incorporates elevated substrate temperature during the spin‐coating stage, as shown in Figure 1a. The increased temperature accelerates solvent evaporation, sharply reducing the time window for QDs’ lateral movement. As a result, QDs solidify rapidly into a disordered yet highly compact film. In this case, the post‐deposition annealing step is primarily responsible for healing local defects generated during rapid solidification, while preserving the overall dense morphology established during deposition. This fundamental difference in the film formation mechanism is illustrated schematically in Figure 1a. Compared to cold spin‐coating, the thermal spin‐coating process integrates fast film formation with targeted defect suppression, producing QD films with improved packing uniformity and lower trap‐state density.

**FIGURE 2:**
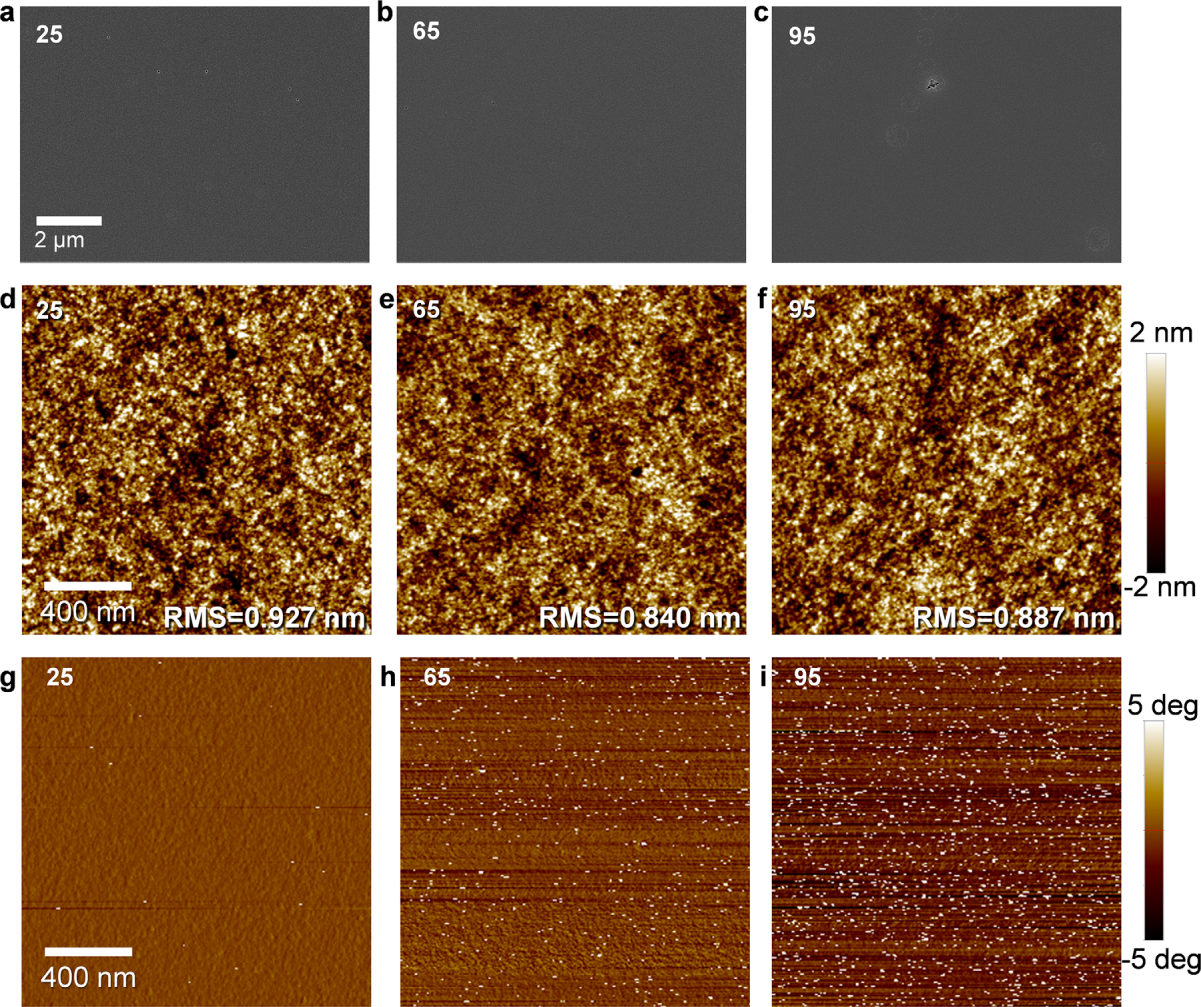
Morphological characterization of PbS QD films prepared by thermal spin‐coating at different substrate temperatures. (a–c) Top‐view SEM images of PbS QD films deposited at 25°C, 65°C, and 95°C, showing continuous and compact film coverage with temperature‐dependent surface features. (d–f) AFM height images of the corresponding films, with RMS roughness values indicated, revealing smooth surfaces with sub‐nanometer roughness. (g–i) AFM phase images highlighting nanoscale packing heterogeneity that becomes more pronounced at elevated substrate temperatures.

Figure S1 shows the TEM image and size histogram of the as‐synthesized PbS QDs, confirming a narrow size distribution with an average diameter of approximately 4.0 nm. The corresponding absorption spectrum (Figure S2) exhibits a well‐defined first excitonic absorption peak at ∼1250 nm, indicating good size uniformity and consistent quantum confinement across the QD ensemble. To investigate the stacking kinetics of QDs under regular deposition and with simultaneous annealing conditions, we performed grazing‐incidence small‐angle x‐ray scattering (GISAXS), and the results are shown in Figure 1b–g. Clear rings of Bragg peaks, primarily stemming from the inter‐dot distance, can be observed in 2D images in Figure 1b–d. The intensity of the scattering peak weakens when the simultaneous annealing temperature is higher, as indicated in Figure 1d. To quantitatively study the internal structure of the QD stackings, we implemented azimuthal integrations, as shown in Figure 2e–g. We used the effective radius of the QDs as the “form factor” and the inter‐dot distance between two neighboring QDs as the “structure factor” to fit the specific range of the integration curves following our previous work [35]. The detailed fitting results are listed in Table S1. Importantly, the effective radius of the QDs is 2.03 ± 0.1 nm, and the inter‐dot distances are 4.33 ± 0.52 nm (t = 25°C), 4.30 ± 0.56 nm (t = 65°C), and 4.29 ± 0.65 nm (t = 95°C). We thus confirmed that when the simultaneous temperature is lower, QDs stack into a relatively ordered configuration with a larger inter‐dot distance. As the simultaneous temperature increases (e.g., beyond 60°C), QDs stack toward a disordered configuration with a smaller inter‐dot distance and a broader distribution width (represented by sigma of D in Table S1), which is due to that the QDs are more compact and more strongly electronically coupled in this condition. Notably, a severe compact can lead to partial fusion of neighboring QDs in solid and further lead to more tail states [12, 36]. The enhanced device performance is thus believed considering the trade‐off between the strong electronic coupling and a narrow energy state distribution.

To further evaluate the morphological differences between films prepared using the two processes, scanning electron microscopy (SEM) and atomic force microscopy (AFM) were conducted on PbS QD films fabricated at 25°C, 65°C, and 95°C (Results for films fabricated at 45°C and 85°C are provided in Figure S3). First, at all processing temperatures, the films exhibit continuous and compact coverage of the substrate without large‐area cracks or pinholes, confirming good film quality. For films deposited at 25°C and 65°C substrate temperatures, the surface appears relatively uniform, with only sparse contrast features distributed across the field of view. As the substrate temperature increases, a slight increase in surface defects can be observed. In particular, the film processed at 95°C shows localized defect‐like or aggregated regions, indicating that excessive thermal energy during deposition affects the film formation process through accelerated solvent evaporation and partial aggregation. Nevertheless, the overall film continuity is largely maintained over the entire temperature range.

Moreover, surface morphology analysis via AFM, shown in Figure 2d–f, highlights differences in surface uniformity among films prepared at different temperatures. The root‐mean‐square (RMS) surface roughness values are 0.927 nm (25°C), 0.840 nm (65°C), and 0.887 nm (95°C), indicating that all films exhibit smooth surfaces with sub‐nanometer roughness. The slightly lower roughness of the 25°C film is consistent with extended lateral mobility of QDs during solvent evaporation, which promotes localized ordering at the surface. In contrast, the film prepared at 65°C shows the lowest RMS roughness and a more homogeneous distribution, reflecting rapid solvent evaporation and dense, uniform QD packing. When the spin‐coating temperature is further increased to 95°C, the RMS roughness increases relative to the 65°C film, accompanied by enhanced local height fluctuations. This behavior indicates that excessive thermal energy during deposition affects the uniformity of the surface packing, consistent with the emergence of localized defect‐like or aggregated regions observed in SEM. While the overall surface remains continuous, the increased roughness at 95°C suggests the onset of thermally induced structural inhomogeneity.

Figure 2g–i shows the AFM phase images of PbS QD films deposited at different substrate temperatures. At 25°C, the phase contrast is weak and narrowly distributed, indicating relatively uniform nanoscale dissipation behavior. With increasing substrate temperature to 65°C, the phase contrast becomes more pronounced, accompanied by localized bright and dark regions, suggesting enhanced heterogeneity in QD packing. At higher temperatures, a significantly increased phase contrast is observed across the scanned area, indicating stronger nanoscale structural variations. Importantly, these changes in phase contrast are not correlated with surface roughness variations, confirming that the observed phase heterogeneity originates from differences in local packing density rather than morphological effects [37, 38].

These structural improvements are critical for suppressing leakage current and promoting uniform carrier transport across the device. These morphological observations confirm that thermal spin‐coating annealing leads to denser, crack‐free QD films without compromising surface smoothness or thickness uniformity. Such compact structures serve as a strong foundation for reducing trap‐assisted recombination and enhancing device performance, as discussed in the subsequent sections. It is noted that, under the present processing conditions (< 95°C), no significant chemical modification of ligands or surface passivation is expected [39]. This is further supported by XPS analysis (Figure S4), where the Br 3d, I 3d, and Pb 4f spectra, as well as the halide‐to‐lead ratios (Table S2), show negligible differences between films prepared at different temperatures. These results indicate that the observed performance improvements mainly originate from structural packing effects rather than variations in surface chemistry.

### 2.2 Electrical Origin of EQE Spectral Shifts

**FIGURE 3:**
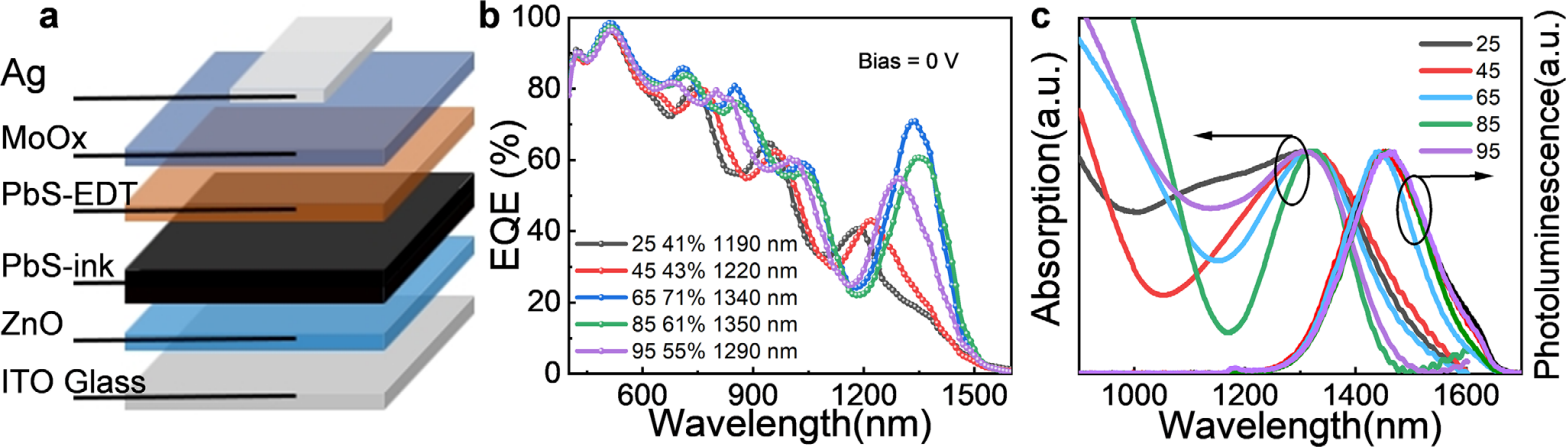
(a) Schematic of the ITO/ZnO/PbS‐ink/PbS‐EDT/MoO_x_/Ag device structure. (b) EQE spectra measured at 0 V bias, showing a maximum EQE at 65°C and a temperature‐dependent peak shift. (c) Normalized absorption and PL spectra of PbS QD films.

Figure 3 summarizes the device structure and spectral characteristics of PbS QD photodetectors fabricated under different spin‐coating temperatures ranging from 25°C to 95°C. As shown in Figure 3a, the device adopts a typical vertical structure composed of an ITO‐coated glass substrate, ZnO electron transport layer, PbS‐ink light absorption layer, PbS‐EDT hole transport layer, MoO_x_ interfacial layer, and Ag top electrode. The use of a bilayer QD configuration, with solution‐phase ligand exchanged PbS‐ink as the active layer and solid‐state ligand exchanged PbS‐EDT as the transport layer, enables efficient carrier separation and extraction. The electron transport layer is sputter‐coated to reach fine crystallizations and uniformity, as shown in Figure S5.

The external quantum efficiency (EQE) spectra of devices prepared at different temperatures are presented in Figure 3b. With increasing spin‐coating temperature from 25°C to 65°C, the EQE exhibits a steady rise in peak intensity, reaching a maximum of 70.8% at 65°C, compared to 40.6% at 25°C. This enhancement reflects improved carrier extraction and suppressed recombination as a result of denser film morphology and better interfacial quality. However, further increasing the temperature to 85°C and 95°C leads to a reduction in EQE, suggesting the presence of thermally induced film defects or interfacial degradation at higher temperatures.

In addition to intensity enhancement, the EQE spectra also exhibit a redshift in the peak position as the spin‐coating temperature increases from 25°C to 65°C, followed by a blueshift at 95°C. Similar EQE peak shifts have been reported in previous studies and are often attributed to a combination of factors, including changes in thickness of the active layer, optical interference conditions, energy level alignment, or wavelength‐dependent carrier extraction efficiency across different layers of the device stack [11, 40, 41].

To investigate the origin of observed changes in EQE, photoluminescence (PL) and absorption spectra were collected for PbS QD films fabricated under the same range of spin‐coating temperatures. As shown in Figure 3c, all PL spectra exhibit nearly identical peak positions centered at ∼1450 nm, with only limited variation across different processing temperatures (Table S3). This indicates that the intrinsic bandgap of the PbS QDs remains essentially unchanged. In addition, cross‐sectional SEM analysis of QD film and fabricated devices (Figures S6 and S7) confirms that the film thickness is nearly identical for different processing temperatures, suggesting that thickness‐dependent optical interference is not the dominant factor. Ultraviolet photoelectron spectroscopy (UPS) measurements (Figures S8 and S9) further show that the overall energy‐level alignment of the PbS QD films remains largely unchanged. Taken together, these results suggest that the observed EQE peak shift is unlikely to originate from changes in intrinsic optical properties or film thickness. Instead, it is more likely influenced by electrically related factors, such as variations in internal electric field distribution, interfacial band bending, and carrier extraction dynamics.

### 2.3 Thermal Spin‐Coating Enabled Enhanced Photodetector Performance

**FIGURE 4:**
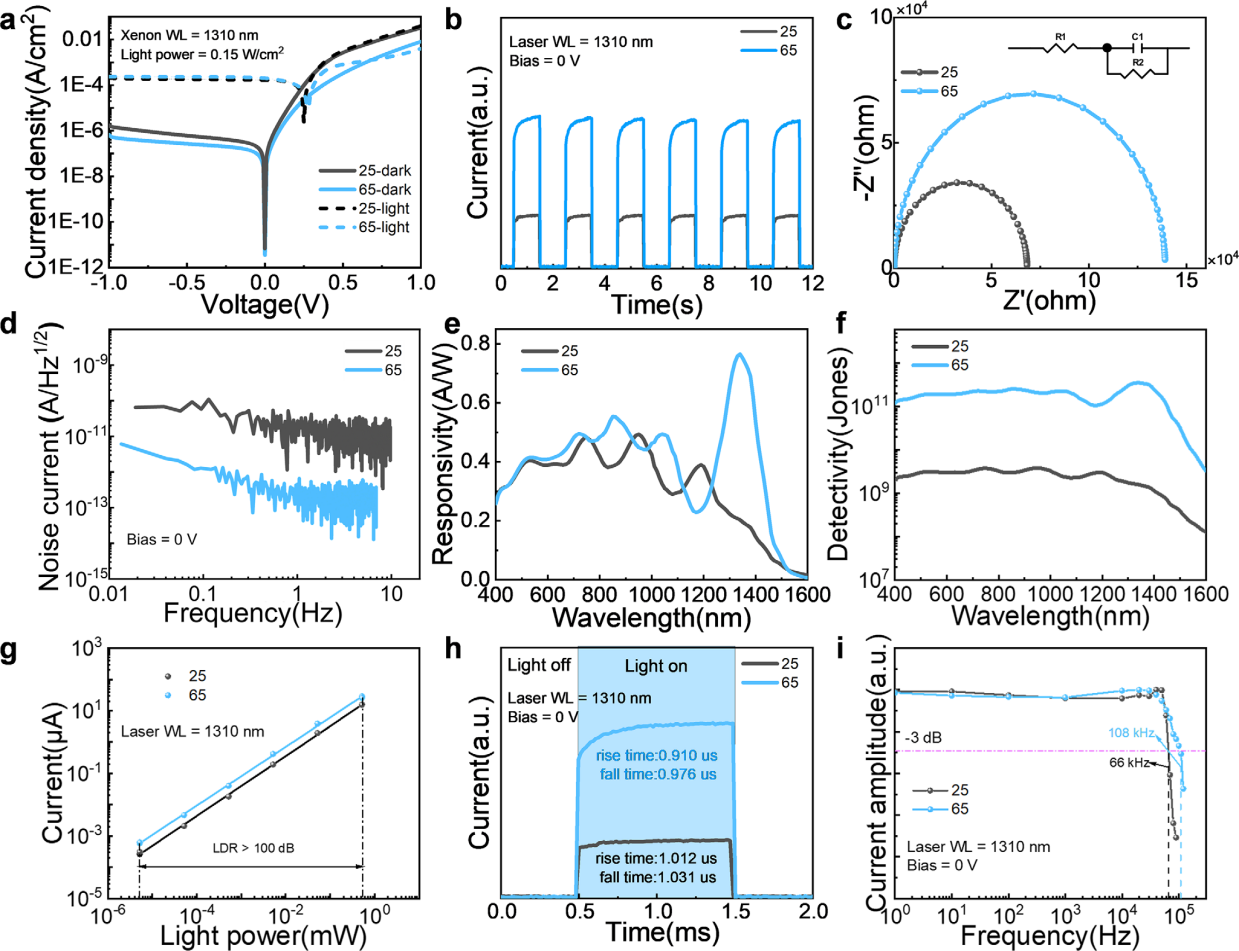
(a) Dark and illuminated *J–V* characteristics under 1310 nm illumination. (b) Steady‐state on/off photocurrent response at zero bias. (c) Nyquist plots obtained from impedance spectroscopy with the equivalent circuit model inset. (d) Low‐frequency current noise spectral density measured at 0 V bias. (e) Spectral responsivity. (f) Specific detectivity as a function of wavelength. (g) Linear dynamic range evaluated at 1310 nm. (h) Transient photoresponse under modulated 1310 nm illumination. (i) Frequency‐dependent photocurrent amplitude and corresponding −3 dB bandwidth.

As shown in Figure 4a, the *J–V* curves under dark and illuminated conditions reveal a clear dual improvement: in addition to a significant suppression of dark current, the 65°C device also exhibits a markedly enhanced photocurrent under 1310 nm illumination. Specifically, at −0.5 V bias, the dark current density drops from 609 nA/cm^2^ (25°C) to 274 nA/cm^2^ (65°C), while the photocurrent increases by nearly an order of magnitude. This indicates that the thermal spin‐coating process not only reduces defect‐related leakage pathways but also enhances the generation and extraction of photocarriers. Such simultaneous optimization of both dark and photocurrent performance addresses two key bottlenecks that limit QD photodetector sensitivity and dynamic range. Further comparison of *J–V* results for devices fabricated under all temperatures are given in Figure S10.

Figure 4b confirms the enhanced photocurrent performance through the steady‐state photoresponse under periodic 1310 nm illumination at zero bias. The device fabricated at 65°C exhibits a higher and stable photocurrent amplitude compared to the 25°C device, indicating more efficient photocarrier generation and extraction. Notably, the relative photocurrent levels are fully consistent with the corresponding *J–V* characteristics shown in Figure 4a, confirming that the improvements in film quality induced by thermal spin‐coating directly translate into enhanced steady‐state optoelectronic response at the device level.

Impedance spectroscopy results (Figure 4c) provide additional insight into the interfacial charge transport behavior. The 65°C device exhibits a much larger semicircle in the Nyquist plot, corresponding to a recombination resistance (R_2_) of 6.3 × 10^5^ Ω, compared to only 3.2 × 10^3^ Ω in the 25°C device. Detailed fitting results is shown in Table S4. This substantial increase in R_2_ indicates more effective carrier separation and reduced interfacial recombination, consistent with improved energy‐level alignment and compact morphology enabled by thermal spin‐coating.

Low‐frequency noise spectral density, shown in Figure 4d, further demonstrates the benefit of the optimized process. The noise current spectral density of the 65°C device is suppressed by nearly two orders of magnitude relative to the 25°C device under 0 V bias, highlighting a significant reduction in defect‐related trap states. Extended‐frequency noise analysis, including comparison with the shot‐noise limit, is provided in Figure S11. This noise suppression is critical for improving the signal‐to‐noise ratio and enabling accurate signal readout in low‐light environments.

The responsivity (*R*) of the device is calculated by the ratio of extracted photocurrent density *J_ph_* and light power density *L_light_* as shown in Equation (1):

(1) $$R=\frac{J_{ph}}{L_{light}}=\frac{I_{light}-I_{dark}}{P_{hv}}\;$$

where *I_light_* and *I_dark_* are the currents under illumination and dark conditions, respectively, and *P_hv_* is the incident optical power on the device. As shown in Figure 4e, the responsivity of the 65°C device reaches 0.765 A/W at 1310 nm under zero bias, significantly higher than the 25°C device (0.43 A/W). This enhancement directly results from improved light absorption, more efficient carrier extraction, and lower leakage current due to improved film compactness and reduced interfacial recombination.

The specific detectivity is calculated using Equation (2):

(2) $$D^{*}=\frac{{\left(A\Delta f\right)}^{\frac{1}{2}}R_{\lambda }}{i_{n}}\;$$

where *R*_λ_ is the wavelength‐dependent responsivity, *A* is the device area in cm^2^, Δ*f* is the electrical bandwidth, and *i_n_* is the measured noise current. In our case, the device area is 0.04 cm^2^, the Δ*f* is set as 1 Hz, and the *i_n_* is obtained from the noise current spectral density in Figure 4d at 1 Hz. As shown in Figure 4f, the device fabricated at 65°C exhibits a peak D* of 3.57 × 10^1^ ^1^ Jones, a marked improvement over the 25°C device (1.89 × 10^1^ ^1^ Jones). The improvement stems from both the increased photocurrent and the significantly reduced noise current, which is consistent with suppressed trap states confirmed by the time‐resolved photoluminescence (TRPL) results, as shown in Figure S12. The photoluminescence decay curves are fitted with a bi‐exponential function, yielding average carrier lifetimes (t_avg_) of 277.97 ns for the 25°C film and 361.28 ns for the 65°C film. Detailed fitting parameters are shown in Table S5. The longer lifetime in the 65°C film suggests a lower density of non‐radiative trap states, consistent with the observed improvement in both steady‐state performance and transient behavior. After deposition of the PbS‐EDT hole transport layer, the carrier lifetime decreases to 8.58 ns (25°C) and 6.76 ns (65°C). This reduction indicates more efficient carrier extraction across the interface rather than increased recombination, as carriers are rapidly transferred from the PbS‐ink layer to the transport layer. The enhanced carrier lifetime also indicates reduced Shockley–Read–Hall recombination, confirming the structural and electronic benefits of the thermal spin‐coating strategy [42, 43, 44]. Further support is provided by space‐charge‐limited current (SCLC) measurements (Figure S13). The device fabricated at 65°C exhibits a lower trap‐filled limit voltage (V_TFL_ ≈ 0.149 V) compared to the 25°C device (V_TFL_ ≈ 0.265 V), together with a higher current density in the trap‐limited regime. This behavior indicates reduced trap‐limited transport and improved carrier mobility in the thermally processed film. These improvements in responsivity and detectivity are a direct consequence of the reduced recombination and efficient carrier extraction resulting from the optimized film formation kinetics during thermal spin‐coating process.

In addition to high responsivity and low noise, linear dynamic range (LDR) is another key metric for evaluating the practical applicability of photodetectors, especially in imaging and sensing scenarios with varying illumination intensities. The LDR reflects the range over which the output photocurrent maintains a linear relationship with incident light intensity, indicating the detector's ability to function reliably under both weak and strong light conditions. Figure 4g summarizes the LDR characteristics of devices fabricated at 25°C and 65°C. Both devices maintain excellent linearity across more than five orders of magnitude of light intensity, yielding an LDR exceeding 100 dB. This wide LDR is critical for real‐world imaging and sensing applications requiring accurate detection under both low and high optical power conditions. The higher slope of the photocurrent vs. light power curve in the 65°C device further confirms superior responsivity and linearity across the tested range.

Figure 4h evaluates the dynamic photoresponse and recombination characteristics of devices fabricated at 25°C and 65°C, providing further insight into their transient behavior and defect‐related charge dynamics. Under a 1310 nm ns laser light source modulated at 1 kHz, the transient response of device processed at 65°C demonstrates a faster and more symmetric transient behavior, with a rise time of 0.910 µs and a fall time of 0.976 µs. In contrast, the 25°C device exhibits a slower response, with a rise time of 1.012 µs and fall time of 1.031 µs. The reduction in response time indicates that thermal spin‐coating improves carrier extraction speed and suppresses slow recombination processes, enabling the device to operate more effectively in high‐frequency or modulated‐light environments. The improved response speed can be attributed to denser film morphology, reduced trap density, and more favorable interfacial energy alignment. The improvement in temporal response is further reflected in the −3 dB bandwidth performance, as summarized in Figure 4i. The −3 dB bandwidth, defined as the frequency at which the photocurrent amplitude drops to 70.7% of its low‐frequency value, increases from 66 kHz for the 25°C device to 108 kHz for the 65°C device. This ∼63% increase in bandwidth demonstrates the clear advantage of thermal spin‐coating in enabling fast and reliable operation at higher modulation speeds. The broader bandwidth results from the combination of reduced trap density, improved film compactness, and enhanced interfacial transport [45]. To further evaluate the device performance, a comparison with representative PbS QD SWIR photodetectors reported in recent years, as well as commercial InGaAs photodetectors, is summarized in Table S6.

### 2.4 Long‐Term Stability and Aging Behavior of Key Device Parameters

We evaluate the long‐term storage stability of the devices under vacuum‐protected conditions to compare the degradation behavior associated with different fabrication processes. After 9 months of storage under vacuum at room temperature without encapsulation, the devices retain measurable photoresponse, allowing comparison of degradation trends under controlled conditions. The transient response of photodetectors changed from a fast rise to a turn‐on value followed by a slow increase. This change of response behavior indicates extra carrier consumption pathways. Compared with the fresh devices shown in Figure 4h, the transient photoresponse of the aged devices exhibits a fundamentally different dynamic behavior. For the fresh devices, illumination induces a rapid turn‐on followed by a gradual increase in photocurrent, indicating that carrier extraction pathways progressively stabilize under continuous illumination. This behavior suggests that, as photogenerated carrier density increases, internal transport channels become more effective and the apparent carrier mobility gradually improves, consistent with trap filling and reduced transport barriers [46, 47].

**FIGURE 5:**
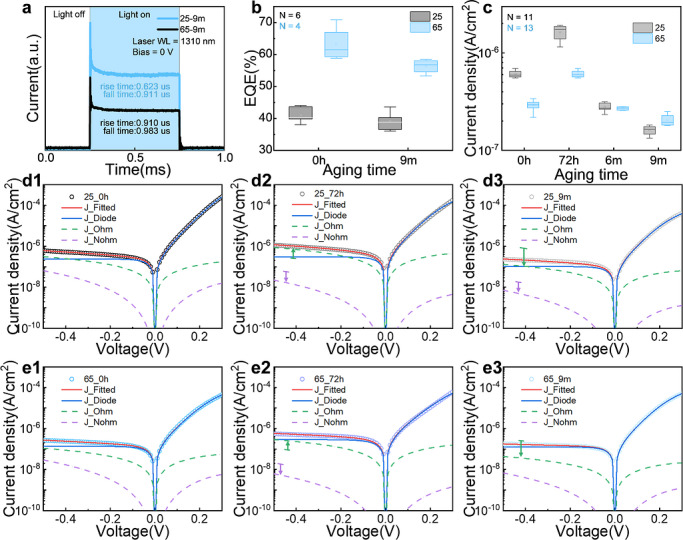
(a) Transient photoresponse of unencapsulated devices after 9 months of storage, showing faster response in both devices compared with fresh devices. (b) Statistical comparison of EQE for fresh devices and after 9 months of aging. (c) Evolution of dark current density at −0.5 V bias as a function of aging time (0 h, 72 h, 6 months, and 9 months). (d1–d3) Dark *J–V* characteristics and component fitting of devices fabricated at 25°C measured at 0 h, 72 h, and 9 months, respectively. (e1–e3) Corresponding dark *J–V* characteristics and fitted current components for devices fabricated at 65°C.

After aging for 9 months under vacuum at room temperature without encapsulation, the transient response of both devices changes markedly, as shown in Figure 5a. Upon illumination, a sharp turn‐on response is first observed, indicating that a large number of photogenerated carriers are initially generated and efficiently extracted, contributing to the fast rising edge of the photocurrent. However, this initial enhancement is followed by a rapid decay of the photocurrent, reflecting the emergence of abundant recombination pathways induced during the aging process. These newly formed recombination centers significantly shorten the effective carrier lifetime, causing photogenerated carriers to recombine before contributing to sustained photocurrent. As illumination continues, the photogenerated carrier density decreases, leading to a reduced recombination rate. The photocurrent then gradually stabilizes at a lower steady‐state value, where the carrier generation rate balances the combined rates of carrier extraction and recombination. In this aged state, slow carriers that previously contributed to the delayed rise in fresh devices are preferentially captured by recombination channels during the turn‐on process and no longer participate in photocurrent generation. As a result, the apparent response speed becomes faster, even though the steady‐state photocurrent is reduced.

The EQE statistics show that devices fabricated at 65°C exhibit a markedly higher initial EQE than those prepared at 25°C. At 0 h, the median EQE is 62% for 65°C devices, compared to 41% for 25°C devices. After 9 months of storage under vacuum without encapsulation, EQE decreases for both cases, but the 65°C devices still retain a higher median EQE (57%) than the 25°C devices (39%), as shown in Figure 5b. This indicates that aging reduces carrier collection efficiency, while the denser films formed at higher spin‐coating temperature better preserve photocarrier extraction. The dark current density (at −0.5 V bias) statistics reveal a distinct aging behavior that differs from the EQE trend as shown in Figure 5c. At the beginning, the 25°C devices exhibit a dark current density of approximately 600 nA/cm^2^, whereas the 65°C devices show a significantly lower value of 290 nA/cm^2^, highlighting the intrinsic leakage suppression enabled by thermal spin‐coating. After 72 h, the dark current increases markedly for both devices, but much more severely for the 25°C samples, which rise to 1300 nA/cm^2^, compared to 610 nA/cm^2^ for the 65°C devices. This pronounced early‐stage increase suggests that films formed at lower temperatures facilitate the generation of additional leakage pathways during initial aging. At longer aging times (6 months and 9 months), the dark current density of both devices decreases and converges to the 100 nA/cm^2^ level. The simultaneous reduction in dark current and degradation in EQE suggests that aging introduces carrier trapping and recombination centers that suppress leakage and extraction, while simultaneously reducing the collection probability of photogenerated carriers. Importantly, devices fabricated at 65°C consistently exhibit lower dark current and higher EQE throughout the aging process, highlighting the superior structural and interfacial stability achieved by thermal spin‐coating. Evolution of *J–V* results are shown in Figure S14.

To better understand the origin of the suppressed dark current, dark current fitting was conducted based on the total current being composed of three components: diode current (J_Diode_), Ohmic leakage current (J_Ohm_), and non‐Ohmic leakage current (J_Non‐ohm_), corresponding to junction‐limited transport, morphology‐related leakage pathways, and trap‐assisted transport, respectively. The fitted curves are shown in Figure 5d,e, and the detailed fitting model is given in the Supporting Information. At −0.5 V bias, fresh devices fabricated at 65°C exhibit a substantially reduced total dark current density (274 nA/cm^2^) compared to those prepared at 25°C (609 nA/cm^2^). This reduction originates primarily from a strong suppression of J_Ohm_, which decreases from 312 nA/cm^2^ (25°C) to 109 nA cm^−^^2^ (65°C), indicating significantly improved film compactness and reduced morphology‐induced leakage. Meanwhile, J_Non‐ohm_ is also lowered from 64.4 to 29.1 nA/cm^2^, suggesting a reduced density of electrically active trap states. In contrast, J_Diode_ shows a more modest decrease (239 to 136 nA/cm^2^), implying that junction‐related transport is less sensitive to the spin‐coating temperature than bulk film quality.

Upon short‐term aging (72 h), both devices exhibit increased dark current, dominated by a pronounced rise in J_Ohm_. Notably, the 25°C device shows a dramatic increase in J_Ohm_ to 866 nA/cm^2^, whereas the 65°C device remains significantly lower at 288 nA/cm^2^. This highlights the superior structural robustness of thermally spin‐coated films. Simultaneously, J_Non‐ohm_ is strongly suppressed in the 65°C device (6.21 nA/cm^2^), indicating improved resistance against trap‐assisted degradation. After long‐term storage (9 months), the advantage of thermal spin‐coating becomes more pronounced. The total dark current of the 65°C device decreases to 177 nA/cm^2^, compared to 239 nA/cm^2^ for the 25°C device. In particular, J_Ohm_ is reduced to only 43.2 nA/cm^2^ in the 65°C device—nearly one‐third of that in the 25°C counterpart—while J_Non‐ohm_ remains low and comparable for both devices (∼7 nA/cm^2^). Detailed fitting parameters and results are given in Tables S7 and S8. These results indicate that thermal spin‐coating not only suppresses initial leakage pathways but also significantly mitigates morphology‐ and trap‐related degradation over time.

Overall, the dark‐current fitting analysis demonstrates that the improved performance and stability of devices fabricated at 65°C are primarily driven by sustained suppression of Ohmic leakage and non‐Ohmic trap‐assisted currents, rather than changes in the intrinsic diode transport. This provides direct electrical evidence that thermal spin‐coating yields dense‐packed, defect‐resilient QD films with long‐term storage stability under vacuum‐protected conditions.

### 2.5 Imaging Chip and Non‐Invasive Glucose Monitoring

Based on enhanced optoelectronic performance, suppressed dark current, and improved long‐term stability enabled by thermal spin‐coating, we further demonstrate the practical applicability of this approach in large‐area imaging and sensing systems. The optimized thermal spin‐coating process at 65°C is employed to fabricate a PbS QD‐based imaging chip and to explore its potential in non‐invasive glucose monitoring.

**FIGURE 6:**
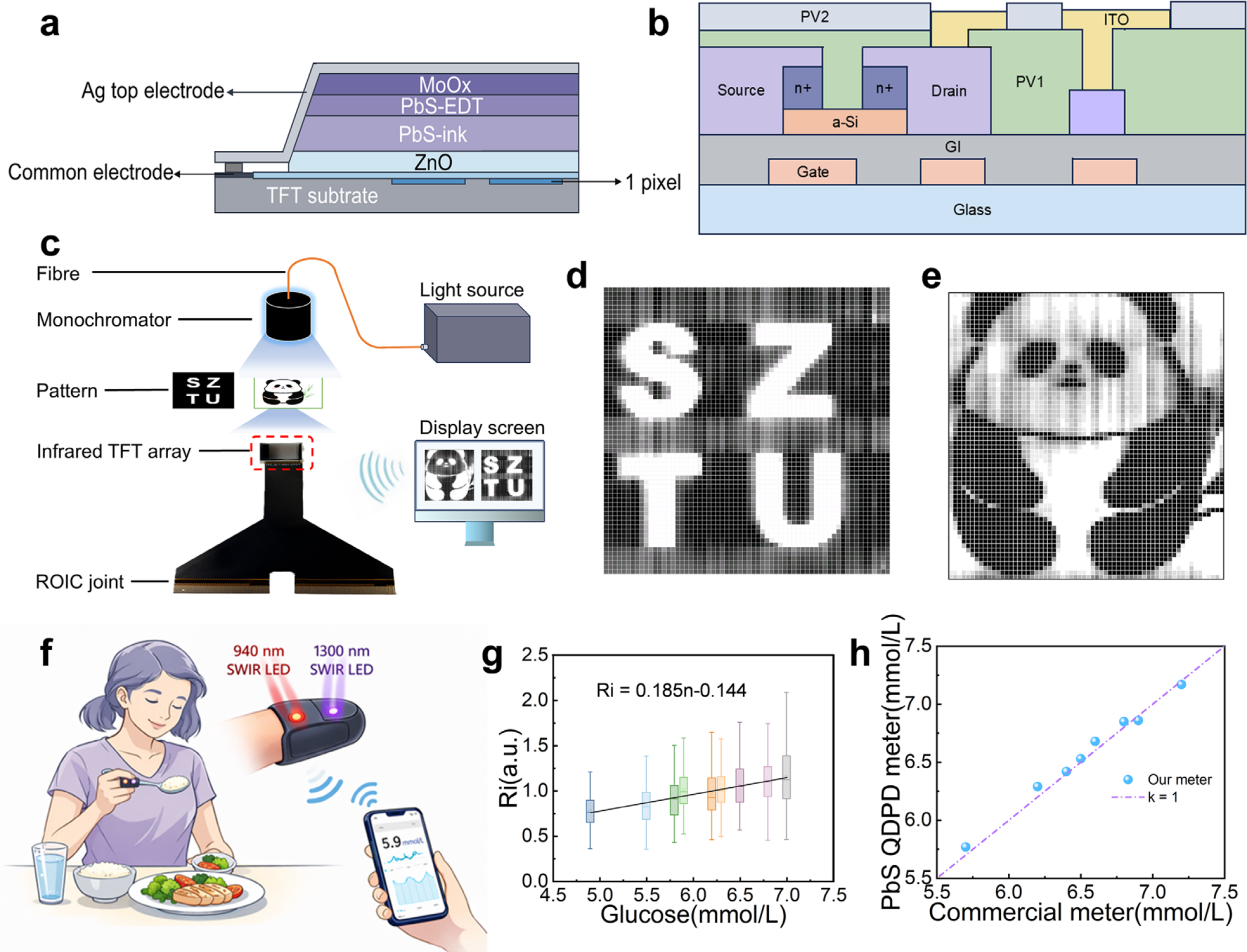
(a, b) Device architecture of a single pixel integrated on a TFT backplane without additional pixel isolation. c Schematic of the 64 × 64 infrared imaging setup and signal collection. (d, e) Reconstructed infrared images showing clear contrast and spatial resolution. (f) Concept of dual‐wavelength (940 and 1300 nm) detection for glucose monitoring. (g) Ratiometric response R_i_ vs. glucose concentration. h Comparison between measured glucose values and a commercial meter.

First, large‐area PbS QD photodetector arrays were directly fabricated on a thin‐film transistor (TFT) backplane, as illustrated in Figure 6a. Owing to the intrinsically low carrier mobility of PbS QD films, the imaging chip fabrication does not require additional pixel isolation steps. Instead, all pixels are fully defined by the underlying TFT electrode array, with the continuous QD film serving as a common photosensitive layer. This electrode‐defined pixel architecture enables straightforward scaling to high pixel densities and offers a distinct advantage over conventional crystalline semiconductor technologies, providing a promising route toward ultrahigh‐resolution imaging chips. Notably, this isolation‐free device architecture is not limited to TFT backplanes and can be readily extended to CMOS substrates, making it particularly attractive for future ultrahigh‐resolution, monolithically integrated imaging systems. In the final fabrication step, a common top electrode is deposited over the pixel area and electrically connected to the pre‐patterned common electrode of the TFT substrate, completing the imager device integration. The detailed structure of the TFT circuit is shown in Figure 6b.

The imaging performance of the PbS QD photodetector array was first evaluated using a custom‐built infrared imaging setup, as illustrated in Figure 6c. A 64 × 64 pixel array fabricated on a TFT backplane was employed, where the incident infrared light was modulated by predefined patterns and projected onto the detector array through a monochromator. The photocurrent signals from each pixel were read out and reconstructed into grayscale images. As shown in Figure 6d,e, the resulting images exhibit clear contrast and well‐resolved features under illumination of 1310 nm light source, demonstrating uniform pixel response and effective spatial definition across the array. The absence of additional pixel isolation, enabled by the electrode‐defined architecture and low carrier mobility of the PbS QD film, does not compromise image clarity, highlighting the suitability of the thermal spin‐coated QD films for high‐resolution infrared imaging applications.

Beyond imaging, the broadband photoresponse of the PbS QD photodetectors enables exploration of their potential in non‐invasive biochemical sensing. As shown in Figure 6f, two wavelengths were selected: 940 and 1300 nm. The wavelength at 940 nm lies within the first near‐infrared biological window (NIR‐I), where optical absorption by water and hemoglobin is minimal and tissue penetration depth is well‐suited for non‐invasive measurements. As a result, the detector response at 940 nm serves as an effective reference channel to suppress physiological background fluctuations and inter‐individual variability. In contrast, 1300 nm falls within the second near‐infrared window (NIR‐II), where glucose exhibits stronger characteristic absorption and light scattering in biological tissue is reduced, thereby enhancing sensitivity to subtle glucose‐induced optical changes [48]. The combination of these two wavelengths enables a balanced detection strategy that simultaneously improves interference rejection and measurement accuracy. By comparing the device responses at these two wavelengths, a ratiometric parameter *R_i_* was defined as the ratio of the photocurrent measured at 1300 nm to that at 940 nm, serving as an indicator correlated with blood glucose concentration, as shown in Equation (3):

(3) $$R_{i}=\frac{{\bar{I}}_{1300}}{{\bar{I}}_{940}}\;$$

where ${\bar{I}}_{1300}$ and ${\bar{I}}_{940}$ are the average photocurrents in 5 frames collected from the TFT readout chip when illuminated by 1300 and 940 nm light, respectively. To ensure data reliability, only stable signal values within 15% ∼ 85% of the full dynamic range of the readout circuit were considered in the analysis, thereby excluding saturated and low‐signal pixels and minimizing the influence of readout noise and non‐uniform pixel response.

Finger‐tip measurements were conducted at different time points after food intake, with glucose concentrations independently measured using a commercial glucose meter for reference. Owing to the 64 × 64 pixel configuration, a large statistical dataset was obtained for each measurement, allowing effective suppression of noise and outlier effects through data averaging and processing (Figure S15). As shown in Figure 6g, the extracted Ri values exhibit a clear linear dependence on glucose concentration over the tested range. Using this linear relationship as a calibration basis, glucose concentrations were estimated from the PbS QD detector responses. Figure 6h shows that the extracted values follow the same trend as those measured by the commercial meter under the tested conditions. This comparison serves as a preliminary consistency check rather than a rigorous clinical validation. Overall, this demonstration highlights the potential of thermally spin‐coated PbS QD photodetector arrays for integrated sensing applications.

## 3 Conclusion

In this work, we demonstrate that thermal spin‐coating annealing is an effective and scalable strategy to improve the performance of PbS quantum dot photodetectors. By elevating the substrate temperature during film deposition, we modulate the solvent evaporation kinetics and quantum dot stacking behavior, resulting in dense and uniform films with suppressed defect density. Comprehensive structural and electrical characterizations confirm that the thermal process enhances interfacial contact, reduces leakage pathways, and facilitates efficient carrier extraction. Devices fabricated at the optimized temperature of 65°C exhibit significantly improved performance metrics, including reduced dark current density, enhanced responsivity (0.765 A/W), higher specific detectivity (3.57 × 10^1^ ^1^ Jones), broader −3 dB bandwidth (108 kHz), and an extended linear dynamic range exceeding 100 dB. Importantly, the optimized devices also demonstrate improved long‐term operational stability, maintaining lower dark current and higher EQE after prolonged storage without encapsulation, which highlights the structural robustness and defect tolerance enabled by thermal spin‐coating. The improvement is achieved without additional passivation layers or material modifications, maintaining compatibility with existing quantum dot ink processes and CMOS integration. Beyond single‐pixel metrics, we further validate the system‐level relevance of this approach by integrating the optimized PbS QD photodetectors onto a TFT backplane to realize a 64 × 64 imaging array. The continuous QD film, together with electrode‐defined pixels, enables array fabrication without additional pixel isolation while maintaining clear image contrast and spatial resolution, supporting a practical route toward high‐density SWIR imaging chips. In addition, benefited from the broadband photoresponse of PbS QDs, we demonstrate a dual‐wavelength ratiometric strategy for non‐invasive glucose monitoring using 940 and 1300 nm, where the extracted ratiometric signal shows a linear correlation with glucose concentration and yields measurement deviations within 5% compared with a commercial glucose meter. Overall, this study highlights the importance of film formation dynamics in determining device performance and provides a simple and reproducible method to simultaneously improve responsivity, response speed, and noise characteristics in colloidal infrared photodetectors. The insights and methodology presented here not only advance the industrial‐scale fabrication of high‐performance SWIR photodetectors but also establish a device‐to‐system pathway toward integrated imaging and wearable biomedical sensing applications.

## 4 Methods

### 4.1 Materials

PbS QDs were synthesized by a standard hot‐injection method. Briefly, 0.45 g of PbO, 8 mL of OA, and 12 mL of ODE were loaded into a 100 mL three‐neck flask, purged with nitrogen at 40°C, and degassed under vacuum at 120°C for 3 h to form a stabilized lead precursor. The sulfur precursor was prepared by dissolving 1 mL of hexamethyldisilthiane ((TMS)_2_S) in 9 mL of ODE. After the lead precursor was cooled to 100°C under nitrogen, 2 mL of the sulfur precursor solution was rapidly injected, and the reaction was maintained for 30–40 s before quenching to room temperature. The crude PbS QDs were purified three times by centrifugation at 7000 rpm for 3 min using n‐hexane and acetone, and the final precipitate was collected for further use.

### 4.2 Precursor Solution Preparations

Liquid‐phase ligand exchange was performed in a glovebox. PbI_2_ (1.844 g, 99.999%, Sinopharm) and PbBr_2_ (0.64 g, 99.999%, Sinopharm) were dissolved in 15 mL of DMF (99.8%, J&K Scientific). The PbS QD precipitate was dispersed in n‐octane (10 mg mL^−^^1^) and transferred into the DMF solution. After vigorous stirring for 3–5 min, the QDs were fully transferred to the DMF phase. The mixture was then allowed to stand for 1–3 min for phase separation, after which the upper organic phase was removed. The DMF phase was washed three times with 10 mL of n‐octane. To precipitate the ligand‐exchanged QDs, 1 mL of n‐octane was added, followed by centrifugation at 7800 rpm for 3 min. The resulting precipitate was dried for 40 min and finally redispersed in a DMF/butylamine mixed solvent at 350 mg mL^−^^1^ for subsequent film deposition.

### 4.3 Film Preparation and Device Fabrications

ITO‐coated glass substrates were cleaned sequentially with deionized water, acetone, and isopropanol under ultrasonication, followed by UV–ozone treatment for 15 min. A 100 nm ZnO electron‐transport layer was deposited by magnetron sputtering. Halide‐exchanged PbS QDs (PbS–PbX_2_, X = I or Br) were then deposited onto the ZnO‐coated substrates by dynamic thermal spin‐coating. Briefly, the substrates were preheated to the desired temperature and maintained for 1 min to ensure thermal stabilization. The PbS ink was drop‐cast onto the rotating substrate and spin‐coated for 40 s, followed by annealing at 85°C for 10 min under nitrogen and cooling to room temperature.

To form the hole‐transport layer, smaller‐sized PbS–PbX_2_ QDs were deposited onto the underlying PbS–PbX_2_ film at 3000 rpm for 30 s, followed by EDT treatment for 30 s and spinning at 4000 rpm for 10 s. The film was subsequently rinsed with acetonitrile at the same spinning speed to remove residual surface ligands. This deposition/ligand‐exchange cycle was repeated twice to obtain two PbS‐EDT layers. The resulting films were oxidized in a desiccator for more than 8 h. Finally, a 10 nm MoO_x_ layer and a 100 nm Ag electrode were sequentially deposited by thermal evaporation to complete the device fabrication.

### 4.4 Characterizations

Absorption spectra of PbS QD films prepared at different spin‐coating temperatures were measured using a near‐infrared spectrometer (NIR 512–1700). Film thickness and surface morphology were characterized by FESEM (Gemini SEM 300) and AFM (Park NX10), respectively. PL and TRPL spectra were recorded using a FluoTime 300 spectrometer. The GISAXS measurements were conducted on an in‐house XEUSS 2.0 SAXS system (Xenocs) with a Cu source (λ = 0.154 nm) and a Pilatus 3R 300K detector. The incidence angle was set to 0.4°, and the sample‐to‐detector distance was 1003 mm. Device performance, including EQE, responsivity, and detectivity, was evaluated using ENLITECH PD‐QE and APD‐QE systems. LDR was characterized using a 1310 nm laser. Noise current spectral density was measured with a Keithley 4200 source meter. Switching characteristics, response time, and −3 dB bandwidth were analyzed using a Tektronix MSO‐24B oscilloscope. The definition of the on/off reference points and the corresponding extraction method are provided in Figure S16. EIS measurements were performed using a CHI 760E electrochemical workstation.

## Author Contributions

**Lei Rao**: data curation, investigation, and formal analysis. **Shuo Cheng**: data curation, investigation, and formal analysis. **Qian Chen**: data curation and investigation. **Jiankai Wang**: data curation and investigation. **Jingrui Ma**: data curation and investigation. **Junjie Hao**: supervision, methodology, and review. **Cun Zheng Ning**: supervision, methodology, and review. **Xiao Wei Sun**: supervision, investigation, and review. **Wei Chen**: investigation, supervision, and writing – original draft. **Haodong Tang**: supervision, investigation, funding acquisition, and writing – original draft.

## Conflicts of Interest

The authors declare no conflicts of interest.

## Supporting information




**Supporting File**: advs75944‐sup‐0001‐SuppMat.pdf.

## Data Availability

The data that support the findings of this study are available from the corresponding author upon reasonable request.

## References

[1] G. Konstantatos , I. Howard , A. Fischer , et al., “Ultrasensitive Solution‐Cast Quantum Dot Photodetectors,” Nature 442 (2006): 180–183, 10.1038/nature04855. 16838017

[2] C. Livache , B. Martinez , N. Goubet , et al., “A Colloidal Quantum Dot Infrared Photodetector and its Use for Intraband Detection,” Nature Communications 10 (2019): 2125, 10.1038/s41467-019-10170-8. PMC6509134 31073132

[3] R. Saran and R. J. Curry , “Lead Sulphide Nanocrystal Photodetector Technologies,” Nature Photonics 10 (2016): 81–92, 10.1038/nphoton.2015.280.

[4] A. J. Houtepen , E. H. Sargent , I. Infante , et al., “Colloidal Quantum Dots for Optoelectronics,” Nature Reviews Methods Primers 5 (2025): 42.

[5] M. A. Hines and G. D. Scholes , “Colloidal PbS Nanocrystals With Size‐Tunable Near‐Infrared Emission: Observation of Post‐Synthesis Self‐Narrowing of the Particle Size Distribution,” Advanced Materials 15 (2003): 1844–1849, 10.1002/adma.200305395.

[6] Y.‐F. Ma , J. Xu , K. Zu , et al., “Breaking the Size Limit of Room‐Temperature Prepared Lead Sulfide Colloidal Quantum Dots for High‐Performance Short‐Wave Infrared Optoelectronics,” ACS Photonics 12 (2025): 1116–1124, 10.1021/acsphotonics.4c02258.

[7] C. Pang , Y.‐H. Deng , E. Kheradmand , et al., “Integrated PbS Colloidal Quantum Dot Photodiodes on Silicon Nitride Waveguides,” Acs Photonics 10 (2023): 4215–4224, 10.1021/acsphotonics.3c00945. 38145169 PMC10741659

[8] H. Wu , Z. Liu , B. Wang , et al., “Integration of PbS Quantum Dots With 3D‐Graphene for Self‐Powered Broadband Photodetectors in Image Sensors,” Acs Photonics 11 (2024): 1342–1351, 10.1021/acsphotonics.3c01803.

[9] S. Zhan , B. Li , T. Chen , et al., “High Responsivity Colloidal Quantum Dots Phototransistors for Low‐Dose Near‐Infrared Photodetection and Image Communication,” Light: Science & Applications 14 (2025): 201, 10.1038/s41377-025-01853-7. PMC12086224 40383854

[10] Y. Yang , H. Zhang , Q. Xue , et al., “High‐Performance Near‐Infrared Computational Spectrometer Enabled by Finely‐Tuned PbS Quantum Dots,” Nano Research 18 (2025): 94907351, 10.26599/NR.2025.94907351.

[11] J. Liu , P. Liu , D. Chen , et al., “A Near‐Infrared Colloidal Quantum Dot Imager With Monolithically Integrated Readout Circuitry,” Nature Electronics 5 (2022): 443–451, 10.1038/s41928-022-00779-x.

[12] L. Chen , S. Liu , Z. Liu , et al., “Complementary Passivation of Bidentate Aromatic Ligands Enables High‐Temperature Stable PbS Colloidal Quantum Dots Image Sensor,” Advanced Functional Materials 35 (2025): 2501770, 10.1002/adfm.202501770.

[13] M.‐T. Jiang , Q. Yang , J.‐L. Xu , et al., “Monolithically Integrated PbS Colloidal Quantum Dot Photodetector Crossbar Array for Short‐Wavelength Infrared Imaging,” Advanced Optical Materials 11 (2023): 2202990, 10.1002/adom.202202990.

[14] X. Wang , Z. Song , H. Tang , et al., “Synergic Surface Modifications of PbS Quantum Dots by Sodium Acetate in Solid‐State Ligand Exchange Toward Short‐Wave Infrared Photodetectors,” ACS Applied Materials & Interfaces 16 (2024): 44164–44173, 10.1021/acsami.4c05201. 39087727

[15] H. Tang , J. Zhong , W. Chen , et al., “Lead Sulfide Quantum Dot Photodetector With Enhanced Responsivity Through a Two‐Step Ligand‐Exchange Method,” ACS Applied Nano Materials 2 (2019): 6135–6143, 10.1021/acsanm.9b00889.

[16] S. Chen , H. Zhong , X. Wang , et al., “Hybrid‐Size Quantum Dots in Hole Transport Layer Depress Dark Current Density of Short‐Wave Infrared Photodetectors,” ACS Photonics 12 (2025): 879–888, 10.1021/acsphotonics.4c01864.

[17] H. Zhu , Z. Yang , H. Xu , et al., “Polyvinylidene Fluoride Enhanced Quantum Dot Short‐Wave Infrared Photodetectors,” IEEE Electron Device Letters 46 (2025): 456.

[18] F. Fang , W. Wang , Y. Li , et al., “Suppressing the Dark Current of PbS QD SWIR Photodetector by Freeze‐Treated Hole Transport Layer,” IEEE Electron Device Letters 46 (2024): 52.

[19] Q. Li , L. Deng , Y. Du , et al., “Interfacial Coupling Enables High Carrier Mobility in PbS Colloidal Quantum Dot Photodetectors,” Nano Research 18 (2025): 94907223.

[20] H. Li , A. Hu , Z. Nie , et al., “A 640×512 ROIC With Optimized BDI Input Stage and Low Power Output Buffer for CQDs‐Based Infrared Image Sensor,” Microelectronics Journal 124 (2022): 105435, 10.1016/j.mejo.2022.105435.

[21] G. Mu , Y. M. Tan , C. Bi , Y. F. Liu , Q. Hao , and X. Tang , “Visible to Mid‐Wave Infrared PbS/HgTe Colloidal Quantum Dot Imagers,” Nature Photonics 18 (2024): 1147–1154, 10.1038/s41566-024-01492-1.

[22] Y. X. Di , K. Ba , Y. Chen , et al., “Interface Engineering to Drive High‐Performance MXene/PbS Quantum Dot Nir Photodiode,” Advanced Science 11 (2023): 2307169. 38044286 10.1002/advs.202307169 PMC10853715

[23] P. Du , J. Li , L. Wang , et al., “Efficient and Large‐Area All Vacuum‐Deposited Perovskite Light‐Emitting Diodes via Spatial Confinement,” Nature Communications 12 (2021): 4751, 10.1038/s41467-021-25093-6. PMC8346511 34362915

[24] Y. Chen , J. Li , X. Yang , Z. Zhou , and C. Q. Sun , “Band Gap Modulation of the IV, III–V, and II–VI Semiconductors by Controlling the Solid Size and Dimension and the Temperature of Operation,” The Journal of Physical Chemistry C 115 (2011): 23338–23343, 10.1021/jp209933v.

[25] J. Luckas , S. Kremers , D. Krebs , M. Salinga , M. Wuttig , and C. Longeaud , “The Influence of a Temperature Dependent Bandgap on the Energy Scale of Modulated Photocurrent Experiments,” Journal of Applied Physics 110 (2011): 013719, 10.1063/1.3605517.

[26] M. Yang , W. Ren , M. Guo , and Y. Shen , “High‐Energy‐Density and High Efficiency Polymer Dielectrics for High Temperature Electrostatic Energy Storage: A Review,” Small 18 (2022): 2205247, 10.1002/smll.202205247. 36266932

[27] Y. Hase , Y. Jadhav , R. Aher , et al., “Annealing Temperature Effect on Structural and Optoelectronic Properties of γ‐In_2_Se_3_ Thin Films Towards Highly Stable Photodetector Applications,” Journal of Molecular Structure 1265 (2022): 133336, 10.1016/j.molstruc.2022.133336.

[28] N. R. Al Amin , C.‐C. Lee , Y.‐C. Huang , et al., “Achieving a Highly Stable Perovskite Photodetector With a Long Lifetime Fabricated via an All‐Vacuum Deposition Process,” ACS Applied Materials & Interfaces 15 (2023): 21284–21295, 10.1021/acsami.3c00839. 37079463

[29] J. Yang , H. Hu , Y. Lv , et al., “Ligand‐Engineered HgTe Colloidal Quantum Dot Solids for Infrared Photodetectors,” Nano Letters 22 (2022): 3465–3472, 10.1021/acs.nanolett.2c00950. 35435694

[30] T. Cao , S. Chen , F. Fang , et al., “Size Effects of 1,2‐Ethanedithiol‐Treated PbS Quantum Dots on Short‐Wave Infrared Photodetector Hole Transport Layers,” The Journal of Physical Chemistry Letters 16 (2025): 4607–4614, 10.1021/acs.jpclett.5c01032. 40311112

[31] J. Wang , L. Rao , L. Xu , et al., “A Search‐and‐Verification Framework for Efficient Annealing Optimization in PbS Quantum Dot Films,” ACS Applied Nano Materials 8 (2025): 13742–13753, 10.1021/acsanm.5c01930.

[32] X. Liu , T. Fu , J. Liu , et al., “Solution Annealing Induces Surface Chemical Reconstruction for High‐Efficiency PbS Quantum Dot Solar Cells,” ACS Applied Materials & Interfaces 14 (2022): 14274–14283, 10.1021/acsami.2c01196. 35289178

[33] S. Y. Zhang , Z. N. Yu , J. Cheng , D. L. Wu , X. Y. Li , and W. Xue , “Effects of Annealing Temperature and Ga Content on Properties of Solution‐Processed InGaZnO Thin Film,” Acta Physica Sinica 65 (2016): 128502.

[34] L. C. Chen , C. C. Chen , J. C. Chen , and C. G. Wu , “Annealing Effects on High‐Performance CH_3_NH_3_PbI_3_perovskite Solar Cells Prepared by Solution‐Process,” Solar Energy 122 (2015): 1047–1051, 10.1016/j.solener.2015.10.019.

[35] W. Chen , R. Guo , H. Tang , et al., “ *Operando*structure Degradation Study of PbS Quantum Dot Solar Cells,” Energy & Environmental Science 14 (2021): 3420–3429, 10.1039/D1EE00832C.

[36] W. Chen , J. Zhong , J. Li , et al., “Structure and Charge Carrier Dynamics in Colloidal PbS Quantum Dot Solids,” The Journal of Physical Chemistry Letters 10 (2019): 2058–2065, 10.1021/acs.jpclett.9b00869. 30964305

[37] H. Wang , Y. Pan , X. Li , et al., “Band Alignment Boosts Over 17% Efficiency Quasi‐2D Perovskite Solar Cells via Bottom‐Side Phase Manipulation,” ACS Energy Letters 7 (2022): 3187–3196, 10.1021/acsenergylett.2c01453.

[38] J. Hu , X. Liu , K. Wang , et al., “A Perylene Diimide Electron Acceptor With a Triphenylamine Core: Promoting Photovoltaic Performance *via*hot Spin‐Coating,” Journal of Materials Chemistry C 8 (2020): 2135–2141, 10.1039/C9TC05713G.

[39] R. C. Keitel , M. C. Weidman , and W. A. Tisdale , “Near‐Infrared Photoluminescence and Thermal Stability of PbS Nanocrystals at Elevated Temperatures,” The Journal of Physical Chemistry C 120 (2016): 20341–20349, 10.1021/acs.jpcc.6b06053.

[40] H. Xia , H. Hu , Y. Wang , et al., “Ligand‐Customized Colloidal Quantum Dots for High‐Performance Optoelectronic Devices,” Journal of Materials Chemistry C 12 (2024): 10919–10928, 10.1039/D4TC01182A.

[41] V. Pejovic , E. Georgitzikis , I. Lieberman , P. E. Malinowski , P. Heremans , and D. Cheyns , “Photodetectors Based on Lead Sulfide Quantum Dot and Organic Absorbers for Multispectral Sensing in the Visible to Short‐Wave Infrared Range,” Advanced Functional Materials 32 (2022): 2201424, 10.1002/adfm.202201424.

[42] S. Zhao and F. Grillot , “Effect of Shockley‐Read‐Hall Recombination on the Static and Dynamical Characteristics of Epitaxial Quantum‐Dot Lasers on Silicon,” Physical Review A 103 (2021): 063521, 10.1103/PhysRevA.103.063521.

[43] P. T. Webster , R. A. Carrasco , A. T. Newell , et al., “Utility of Shockley–Read–Hall Analysis to Extract Defect Properties From Semiconductor Minority Carrier Lifetime Data,” Journal of Applied Physics 133 (2023): 125704, 10.1063/5.0147482.

[44] Y. Jiang , Y. Zhang , J. Zheng , et al., “Infrared PbS Quantum Dot–Lead Halide Perovskite Combinations for Breaking the Shockley–Queisser Limit,” Solar RRL 9 (2025): 2400743, 10.1002/solr.202400743.

[45] U. Bothra , M. Albaladejo‐Siguan , Y. Vaynzof , and D. Kabra , “Impact of Ligands on the Performance of PbS Quantum Dot Visible–Near‐Infrared Photodetectors,” Advanced Optical Materials 11 (2023): 2201897, 10.1002/adom.202201897.

[46] Q. W. Xu , L. J. Meng , K. Sinha , F. Chowdhury , J. Hu , and X. H. Wang , “Ultrafast Colloidal Quantum Dot Infrared Photodiode,” ACS Photonics 7 (2020): 1297–1303, 10.1021/acsphotonics.0c00363.

[47] A. M. Najarian , M. Vafaie , B. Chen , F. P. G. de Arquer , and E. H. Sargent , “Photophysical Properties of Materials for High‐Speed Photodetection,” Nature Reviews Physics 6 (2024): 219–230, 10.1038/s42254-024-00699-z.

[48] J. Yadav , A. Rani , V. Singh , and B. M. Murari , “Prospects and Limitations of Non‐Invasive Blood Glucose Monitoring Using Near‐Infrared Spectroscopy,” Biomedical Signal Processing and Control 18 (2015): 214–227, 10.1016/j.bspc.2015.01.005.

