# Supplementary material for: Thermal Spin Coated PbS QD SWIR Imager for Non‐Invasive Glucose Monitoring
Source: Adv Sci (Weinh). 2026 Jun 1;13(43):e75944. doi: 10.1002/advs.75944 (PMC13337112; doi:10.1002/advs.75944)
Supplement: Supplementary file 1 — Supporting File: advs75944‐sup‐0001‐SuppMat.pdf. [file ADVS-13-e75944-s001.pdf]

Thermal Spin Coated PbS QD SWIR Imager for Non-invasive Glucose Monitoring

Lei Rao[#], Shuo Cheng[#], Qian Chen, Jiankai Wang, Jingrui Ma, Junjie Hao[*], Xiao Wei Sun[*], Wei Chen[*], Cun Zheng Ning[*],Haodong Tang[*],

Author Information

Affiliations

College of Integrated Circuits and Optoelectronic Chips, Shenzhen Technology University, Shen Zhen, China

Lei Rao[#], Shuo Cheng[#], Jiankai Wang, Junjie Hao*, Cun Zheng Ning*, Haodong Tang*

College of Engineering Physics, Shenzhen Technology University, Shen Zhen, China

Qian Chen & Wei Chen*

Institute of Nanoscience and Applications, and Department of Electronic and Electrical Engineering, Southern University of Science and Technology, Shen Zhen, China

Jingrui Ma & Xiao Wei Sun*

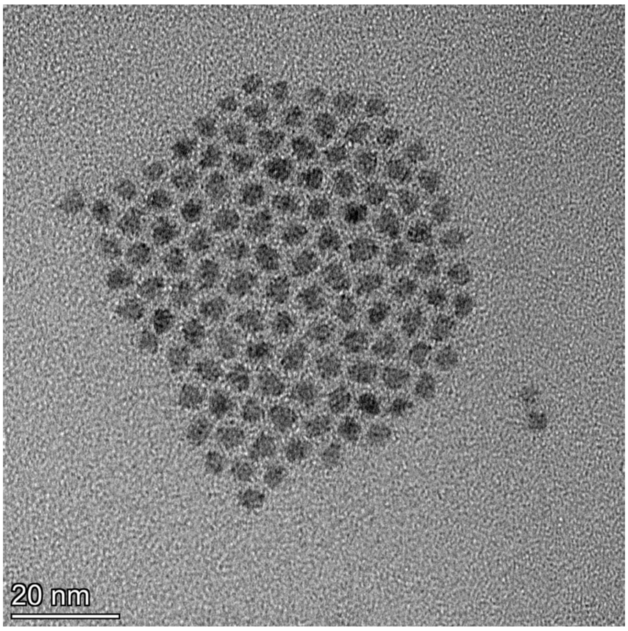

Figure S1. TEM image of the as-synthesized PbS quantum dots and the corresponding size histogram, showing a narrow size distribution with an average diameter of ~4.0 nm.

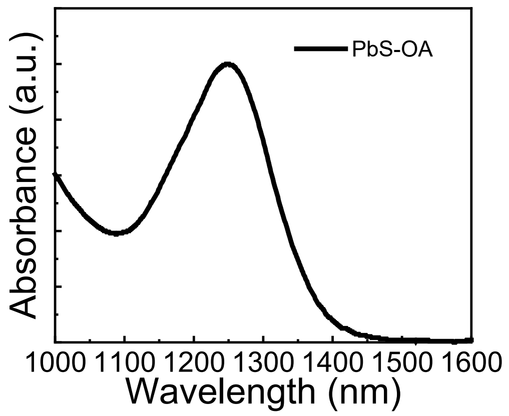

Figure S2. Absorption spectrum of oleic-acid-capped PbS QDs in solution, exhibiting a clear first excitonic feature (~1250 nm), consistent with good size uniformity.

sample	R	sigma	D	sigma
t = 25°C	2.03	0.10	4.33	0.52
t = 65°C	2.03	0.10	4.30	0.56
t = 95°C	2.03	0.10	4.29	0.65

Table S1. GISAXS fitting parameters for PbS QD films deposited at different temperatures, including effective QD radius, inter-dot distance, and the distribution width (σ) of inter-dot spacing.

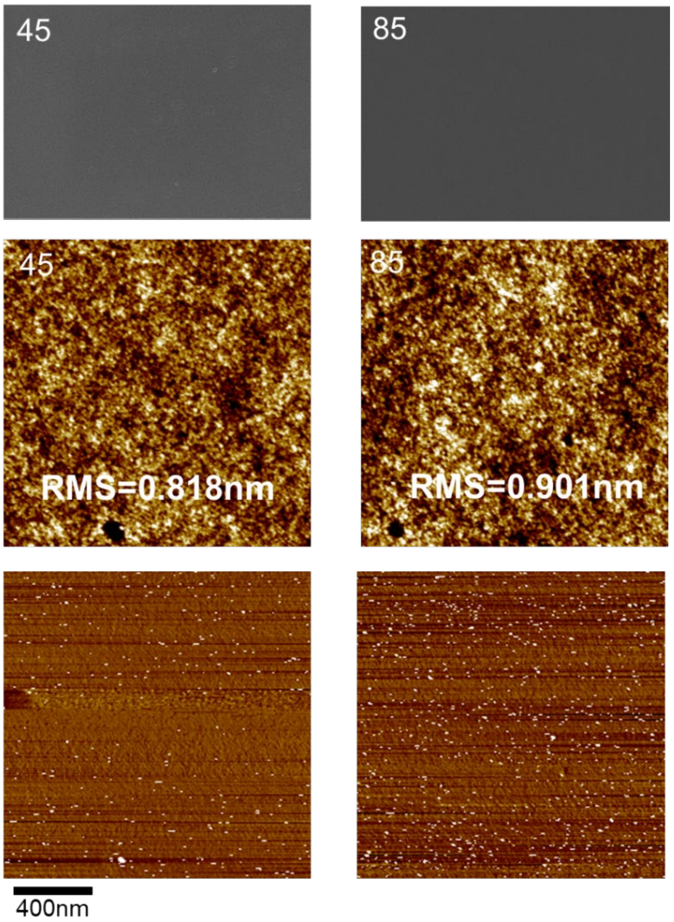

Figure S3. Additional surface morphology of PbS QD films deposited at intermediate temperatures.

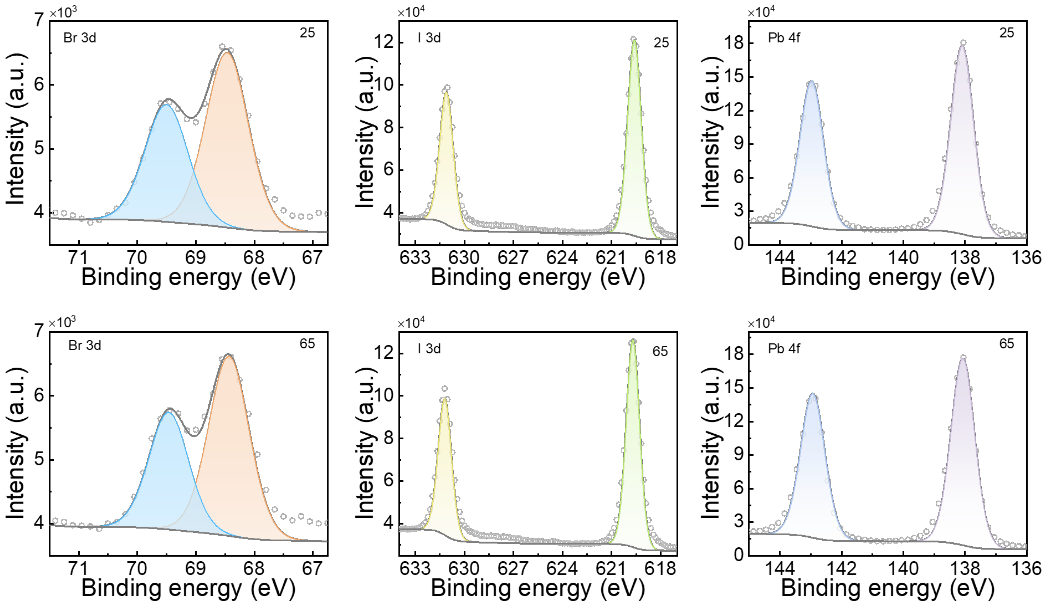

Figure S4. XPS analysis of PbS QD films prepared at different spin-coating temperatures.

Name	25 Atomic %	65 Atomic %	X/Pb (25)	X/Pb (65)
Br 3d	6.42	6.10	0.182	0.169
I 3d	18.06	18.46	0.514	0.517
Br 3d + I 3d	10.74	10.00	0.695	0.685
Pb 4f	32.82	32.98	/	/

Table S2. Quantitative XPS analysis of PbS QD films prepared at 25 °C and 65 °C.

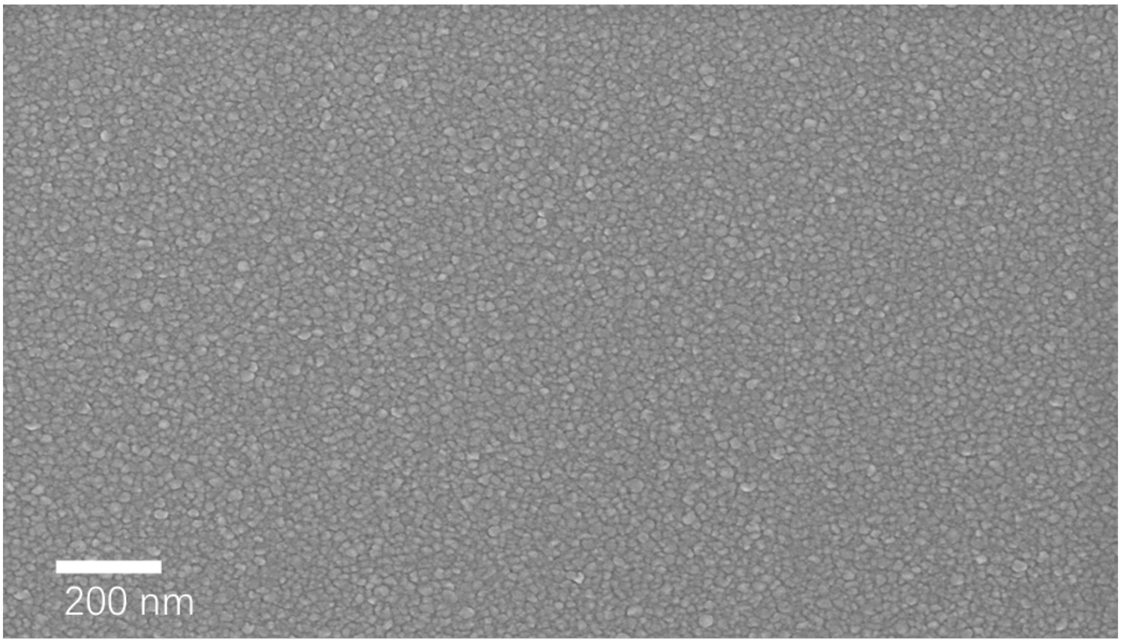

Figure S5. Morphology characterization of the sputtered ZnO electron-transport layer, confirming uniform coverage and good film quality.

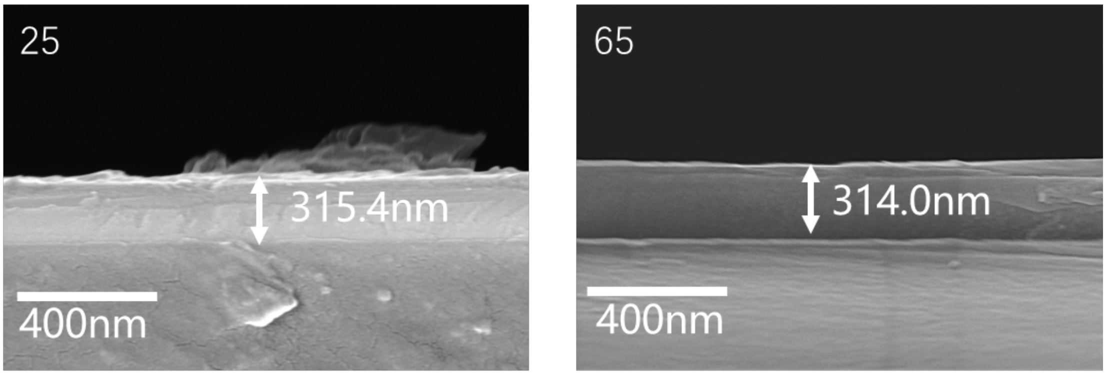

Figure S6. Cross-sectional SEM images of devices/films prepared at 25 °C and 65 °C, showing comparable active-layer thickness (≈315 nm) and uniform vertical stacking.

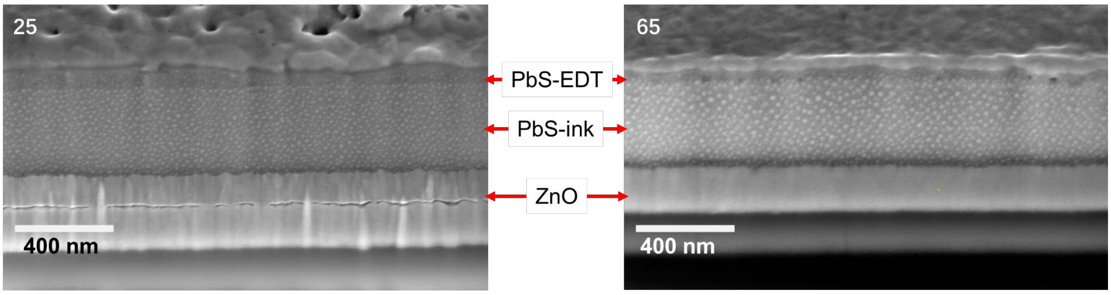

Figure S7. Cross-sectional SEM images of PbS QD photodetector devices fabricated at 25 °C (left) and 65 °C (right). The multilayer structure, consisting of ZnO, PbS-ink, and PbS-EDT layers, is clearly resolved. Both devices exhibit comparable film thicknesses and well-defined layer interfaces, indicating that thermal spin-coating does not significantly alter the overall device architecture. The 65 °C film shows a more uniform and compact morphology, consistent with improved film formation. Scale bars: 400 nm.

**Temperature (°C)**	**Absorbance peak (nm)**	**Photoluminscence peak (nm)**
25	1304	1454
45	1317	1450
65	1319	1442
85	1324	1460
95	1308	1462

Table S3. Extracted peak positions from absorption and PL spectra for PbS QD films prepared at different spin-coating temperatures.

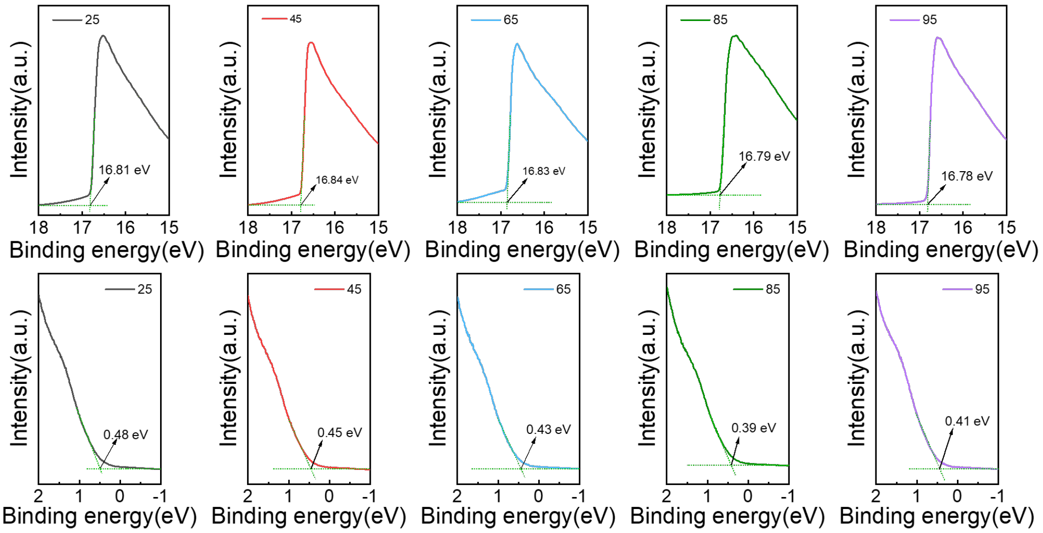

Figure S8. UPS spectra of PbS QD films prepared under different spin-coating temperatures. Including secondary electron cutoff region for work-function extraction and valence-band region for valence-band maximum determination.

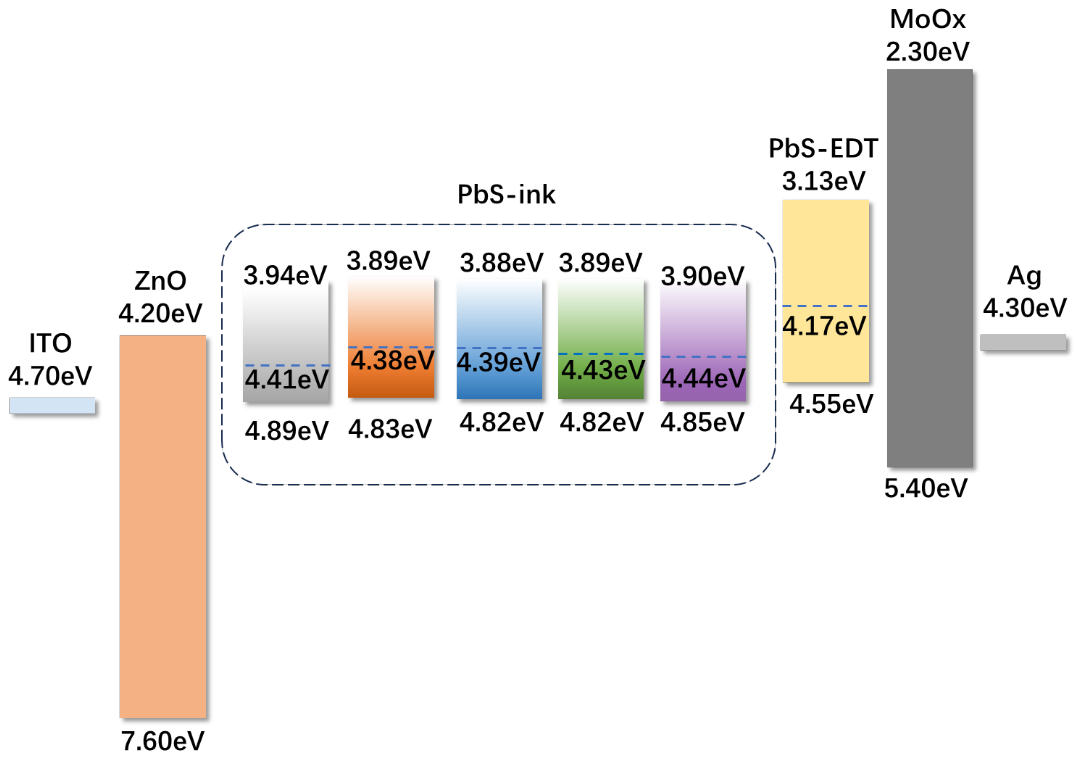

Figure S9. Energy-level alignment of PbS QD films/devices derived from UPS and energy bandgap analysis, showing negligible variation in work function and band structure with spin-coating temperature.

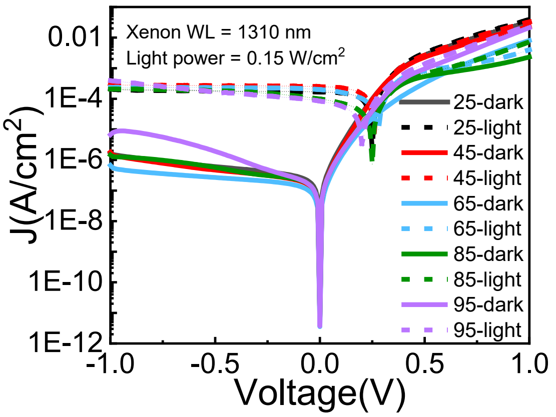

Figure S10. Dark and illuminated J–V characteristics of PbS QD photodetectors fabricated at different thermal spin-coating temperatures (25–95 °C), showing temperature-dependent dark-current suppression and photocurrent enhancement.

**Temperature(°C)**	**R_1(ohm)**	**C(F)**	**R_2(ohm)**
25	0.24	2.9e-9	3.2e3
65	0.30	2.8e-9	6.3e5

Table S4. Equivalent-circuit fitting results from impedance spectroscopy (e.g., R_1, R_2, C), comparing devices fabricated at 25 °C and 65 °C.

Noise Analysis at Extended Frequency Range

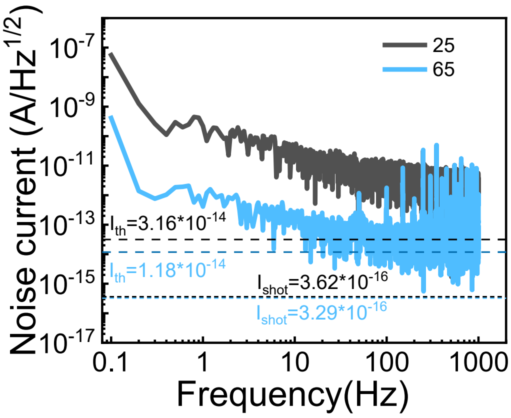

Figure S11. Noise current spectral density of PbS QD photodetectors fabricated at 25 °C and 65 °C measured over an extended frequency range under 0 V bias.

To further evaluate the noise characteristics of the devices, the current noise spectral density was measured over an extended frequency range. At low frequencies, the noise is dominated by 1/f noise, which is associated with trap-assisted carrier fluctuations in the PbS QD film. As the frequency increases, the noise spectrum gradually approaches a frequency-independent regime.

For comparison, the shot-noise limit was calculated using i_shot=√(2qI_dark), where $q$is the elementary charge and $I_{dark}$is the dark current. The measured noise of the optimized device approaches the shot-noise limit at higher frequencies, suggesting that trap-related noise is effectively suppressed under these conditions. Importantly, the overall noise behavior remains consistent with the main text, confirming that the reduced noise level in devices fabricated at 65 °C originates from suppressed trap states and improved film quality, rather than measurement artifacts. The inclusion of the extended frequency range and shot-noise comparison further validates the robustness of the noise analysis.

Time-Resolved Photoluminescence (TRPL) Analysis

The TRPL decay curves were fitted using a bi-exponential function:

$$y = y_0 + A_1 * e^{-\frac{x}{\tau_1}} + A_2 * e^{-\frac{x}{\tau_2}} \quad \text{(S1)}$$

where y_0 is the background offset, A_1 and A_2 are the relative amplitudes, and τ_1 and τ_2 represent the fast and slow decay lifetimes, respectively. The fast component is typically associated with trap-assisted or non-radiative recombination, while the slow component corresponds to radiative recombination of band-edge carriers. To quantitatively compare carrier recombination dynamics, an amplitude-weighted average lifetime was calculated as:

$$\tau_{avg} = \frac{A_1 * \tau_1^2 + A_2 * \tau_2^2}{A_1 * \tau_1 + A_2 * \tau_2} \quad \text{(S2)}$$

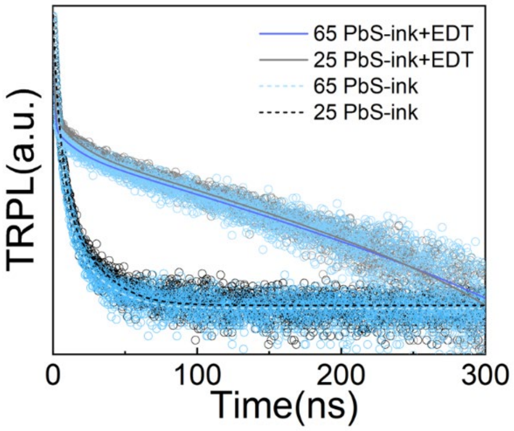

Figure S12. Time-resolved photoluminescence (TRPL) decay curves of PbS QD films prepared at 25 °C and 65 °C with bi-exponential fitting; the 65 °C film exhibits a longer average lifetime, indicating reduced non-radiative recombination.

**Temperature(°C)**	**A_1**	**τ_1(ns)**	**A_2**	**τ_2(ns)**	**τ_{avg}(ns)**
25 PbS-ink	416	300.16	422.4	26.04	277.97
65 PbS-ink	341	393.5	334.2	40.3	361.28
25 PbS-ink+EDT	1430.1	2.701	159	16.98	8.58
65 PbS-ink+EDT	1396.1	2.336	170.9	13.177	6.76

Table S5. TRPL bi-exponential fitting parameters and derived average lifetimes (t_{avg}) for PbS QD films prepared at 25 °C and 65 °C.

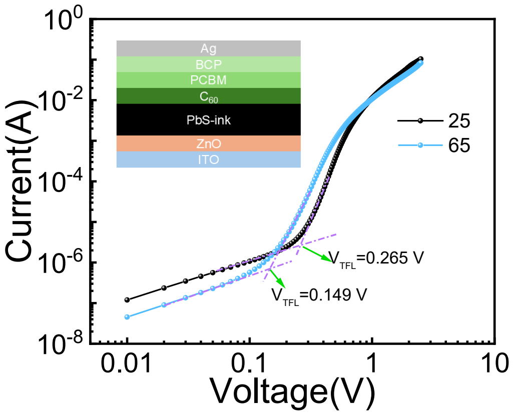

Figure S13. Space-charge-limited current (SCLC) characteristics of electron-only devices based on PbS QD films prepared at 25 °C and 65 °C. The device structure is illustrated in the inset. The current–voltage curves show a transition from the ohmic region to the trap-filled limit (TFL) regime, from which the trap-filled limit voltage (V_TFL) is extracted. The device fabricated at 65 °C exhibits a lower V_TFL and higher current density, indicating reduced trap density and enhanced carrier mobility compared to the 25 °C device.

Device structure	Wavelength (nm)	Responsivity (A/W)	Detectivity (Jones)	Dark current density (A/cm^2)	f_{-3dB} (Hz)	LDR (dB)	Year	Ref
ITO/ZnO/PbS-X/PbS-EDT/MoOx/Ag	1310	0.765@0 V	3.57×10^{11}@0 V	2.74×10^{-7}@-0.5 V	108k	>100	/	This work
ITO/ZnO/PbS-TBAI/PbS-EDT/Au	1330	0.8@-1 V	1.8×10^{12}@0 V	1.8×10^{-6}@−1 V	75k	/	2024	S1
ITO/NiO_x/PbS-EDT/CuInSeS/ZnO/Al	940	0.23@-1 V	1.87×10^{12}@−1 V	4.60×10^{-8}@−1 V	/	109.1	2024	S2
ITO/SnO_2/PbS QD/Au	2100	0.14	4.0×10^{11}	1.6×10^{-7}@−1.0 V	/	/	2024	S3
ITO/PEIE/ZnO/PbS QD/Poly-TPD/MoO_3/Ag	1413	0.47@−0.5 V	2.22×10^{12}@−0.5 V	1.28×10^{-8}@−0.5 V	/	/	2022	S4
ITO/ZnO/PbS-X/PbS-EDT/MoO_x/Ag	1320	/	8.08×10^{10}@0 V	8.55×10^{-7}@−0.5 V	/	/	2025	S5
ITO/NiO_x/PbS-EDT/SAM/PbS-X/C_{60}/PCBM/BCP/Ag	1200	~0.5@0 V	1.64×10^{12}@0 V	2.20×10^{-7}@−0.5 V	62k	92	2025	S6
ITO/ZnO/QD ink/QD-HTL/MoO_x/Ag	~1200	0.54@0 V	5.50×10^{12}@0 V	3.22×10^{-7}@−0.5 V	/	/	2025	S7
ITO/SnO_2/PbS(active)/PbS-EDT/Au	1650	/	8.73×10^{12}	2.1×10^{-8}@−0.5 V	/	103	2026	S8
Hamamatsu G12180-003A (InGaAs)	1550	1.1	6.3×10^{12}	1.4×10^{-7}	600M	/	2024	/
Hamamatsu G12180-210A (InGaAs)	1550	/	6.7×10^{13}	1.3×10^{-9}	40M	/	2024	/
Excelitas C30619GH (InGaAs)	1300/1500	0.90/0.95	/	1.5×10^{-7}	350M	/	2023	/

Table S6. Comparison of device performance for representative PbS QD SWIR photodetectors in recent years and commercial InGaAs photodetectors.

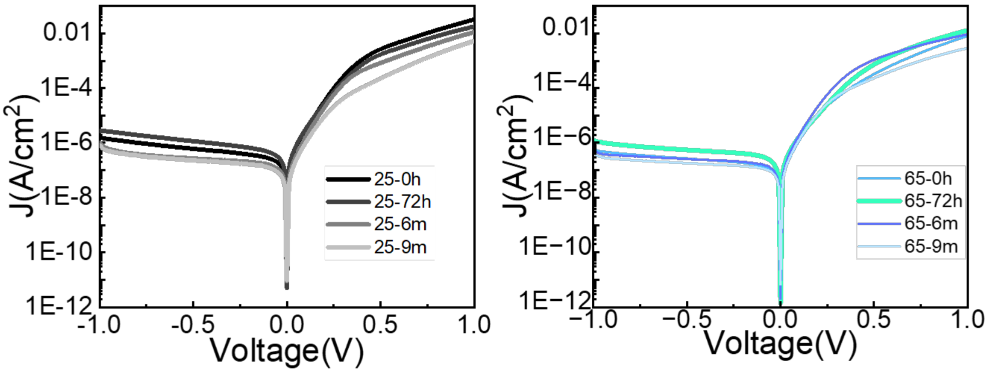

Figure S14. Evolution of dark J–V characteristics during storage/aging (0 h, 72 h, 6 months, and 9 months) for devices fabricated at 25 °C and 65 °C, demonstrating distinct short-term increase and long-term reduction of leakage.

Dark-current J–V fitting model and equations

To quantify the physical origins of dark current, the measured dark current density is decomposed into three additive contributions:

$$J_D = J_0\left(e^{\frac{e}{nk_BT}(V-J_DR_s)} - 1\right) + \frac{V - J_DR_S}{R_{SH}} + k(V - J_DR_S)^m \qquad \text{(S3)}$$

1) Diode (junction-limited) current

The junction-related component is described by the Shockley diode equation:

$$J_{\text{Diode}}(V) = J_0\left(e^{\frac{e}{nk_BT}(V-J_DR_s)} - 1\right) \qquad \text{(S4)}$$

where $J_0$is the reverse saturation current density, $n$is the ideality factor, $q$is the elementary charge, $k_B$is the Boltzmann constant, and $T$is temperature. This term primarily reflects transport governed by the diode junction and interfacial band bending.

2) Ohmic leakage (shunt-like) current

Morphology-induced leakage (e.g., pinholes, microcracks, local shunts) is captured by a linear Ohmic term:

$$J_{\text{Ohm}}(V) = \frac{V - J_DR_S}{R_{SH}} \qquad \text{(S5)}$$

where $R_{\text{sh}}$is the shunt resistance and R_S is the series resistance. A lower J_{Ohm}indicates improved film compactness and fewer morphology-related leakage pathways.

3) Non-Ohmic leakage (trap-assisted) current

The remaining non-linear leakage is assigned to trap-assisted transport (field-enhanced hopping/tunneling through gap states). It can be described by a symmetric field-activated form:

$$J_{\text{Non-ohm}}(V) = k\left(V - J_DR_S\right)^m \qquad \text{(S6)}$$

where $k$is a fitting coefficient related to the density of electrically active trap states, and $m$is the exponent characterizing the nonlinear, field-dependent transport behavior. This current component reflects trap-assisted hopping or field-enhanced carrier emission within the QD solid and is distinct from morphology-related Ohmic leakage. A reduced $J_{\text{Non-ohm}}$therefore indicates effective suppression of trap-mediated leakage pathways.

Parameters	25	65	25-72h	65-72h	25-9m	65-9m
J_0(nA/cm^2)	239	136	304	286	102	127
R_S(Ω)	8.10×10^1	1.04×10^3	2.94×10^2	5.73×10^2	1.39×10^3	1.04×10^3
R_{SH}(Ω)	1.60×10^6	4.58×10^6	5.77×10^5	1.73×10^6	3.84×10^6	1.98×10^7
k	3.40×10^{-7}	1.54×10^{-7}	1.17×10^{-7}	3.28×10^{-8}	3.76×10^{-8}	1.77×10^{-7}

Table S7. Fitting parameters of dark current for devices fabricated at 25 °C and 65 °C at different aging times.

J(nA/cm^2)	25	65	25-72h	65-72h	25-9m	65-9m
J_D	609	274	1190	581	239	177
J_{Diode}	239	136	304	286	102	127
J_{Ohm}	312	109	866	288	130	43.2
$J_{\text{Non-ohm}}$	64.4	29.1	22.2	6.21	7.11	6.75

Table S8. Decomposed dark-current components at −0.5 V (total current J_D, diode current J_{Diode}, Ohmic leakage J_{Ohm}, and non-Ohmic leakage $J_{\text{Non-ohm}}$) for devices fabricated at 25 °C and 65 °C at different aging times.

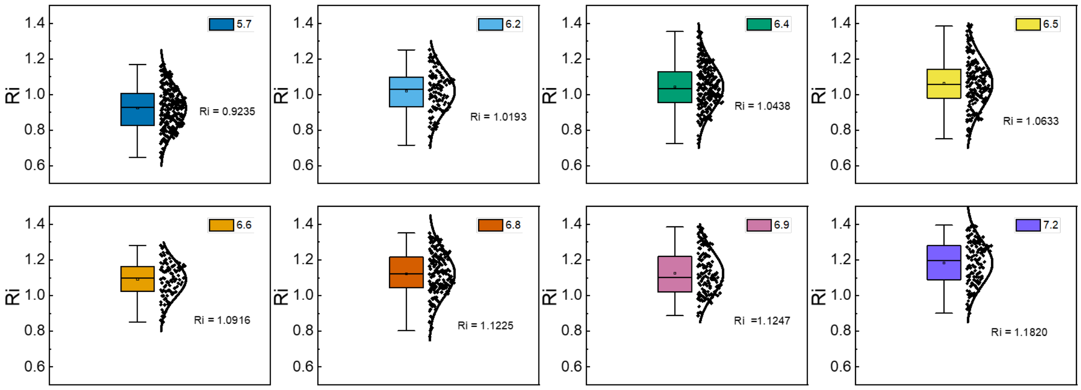

Figure S15. Statistics for ratiometric glucose monitoring using the 64 × 64 TFT readout: pixel-level signal filtering/selection, frame averaging (5 frames), and extracted distribution of R_i under 940 nm and 1300 nm illumination.

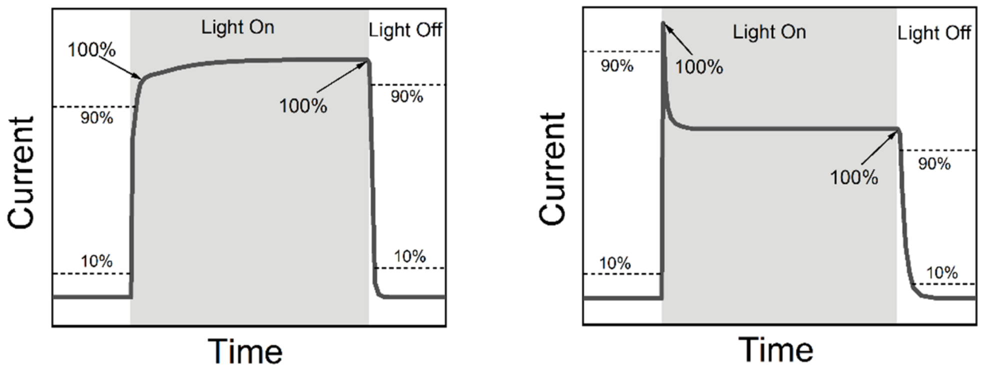

Figure S16. Schematic illustration of the definition of response time for different transient behaviors in PbS QD photodetectors. Left: fresh device, showing a rapid turn-on followed by a gradual increase toward a steady-state current. Right: aged device, exhibiting an initial overshoot peak followed by relaxation to a lower steady-state level. For the rising edge, the 100% reference is defined at the characteristic transition point after turn-on (onset of slow rise for fresh devices and peak of overshoot for aged devices), and the response time is extracted between 10% and 90% of this reference. For the falling edge, the steady-state current under illumination is defined as 100%, and the decay time is extracted from 90% to 10%.

Reference

S1. Wang H*, et al.* PbS quantum dots ink with months-long shelf-lifetime enabling scalable and efficient short-wavelength infrared photodetectors. *Advanced Materials* **36**, 2311526 (2024).

S2. Chen Z*, et al.* Suppression of the dark current in PbS quantum dot infrared photodetectors through the introduction of a CuInSeS interfacial layer. *Journal of Materials Chemistry C* **12**, 4493-4500 (2024).

S3. Wang Q*, et al.* Size-controllable fabrication of PbS quantum dots for NIR–SWIR photodetectors with extended wavelengths. *Journal of Materials Chemistry C* **12**, 19595-19602 (2024).

S4. Yuan Y, Xu J-L, Zhang J-Y, Gao X, Zhong Y-N, Wang S-D. Interface engineering for high photoresponse in PbS quantum-dot short-wavelength infrared photodiodes. *IEEE Electron Device Letters* **43**, 1275-1278 (2022).

S5. Fang F*, et al.* Interface-Enhanced PbS Quantum Dot Short-Wave Infrared Photodetector toward Instant Smoke Detection Applications. *ACS Applied Electronic Materials* **7**, 7368-7376 (2025).

S6. Chen S*, et al.* Self-assembled monolayer-modified hole transport layers for high-performance CMOS-compatible PbS quantum dot photodetectors. *Nano Research* **18**, 94907796 (2025).

S7. Cao T*, et al.* Size Effects of 1, 2-Ethanedithiol-Treated PbS Quantum Dots on Short-Wave Infrared Photodetector Hole Transport Layers. *The Journal of Physical Chemistry Letters* **16**, 4607-4614 (2025).

S8. Xia H, Lv L, Ran X, Yuan M, Lan X. Ultralow Dark Current and Broadband PbS Colloidal Quantum Dot Photodetectors. *ACS Photonics*, (2026).
